# Salt-inducible kinases inhibitor HG-9-91-01 targets RIPK3 kinase activity to alleviate necroptosis-mediated inflammatory injury

**DOI:** 10.1038/s41419-022-04633-y

**Published:** 2022-02-25

**Authors:** Dongxuan Huang, Pengfei Chen, Guoqing Huang, Huimin Sun, Xiaohua Luo, Chaowen He, Fei Chen, Yong Wang, Changchun Zeng, Lianhui Su, Xiaobin Zeng, Jiachun Lu, Shiyue Li, Dongsheng Huang, Hanchao Gao, Mengtao Cao

**Affiliations:** 1 Department of Respiratory Medicine, Shenzhen Longhua District Central Hospital, Affiliated Central Hospital of Shenzhen Longhua District, Guangdong Medical University, Shenzhen, 518110 China; 2 The State Key Lab of Respiratory Disease, The First Affiliated Hospital, The Institute for Chemical Carcinogenesis, School of Public Health, Guangzhou Medical University, Guangzhou, 510182 China

**Keywords:** Necroptosis, Target validation

## Abstract

Receptor-interacting protein kinase 3 (RIPK3) functions as a central regulator of necroptosis, mediating signaling transduction to activate pseudokinase mixed lineage kinase domain-like protein (MLKL) phosphorylation. Increasing evidences show that RIPK3 contributes to the pathologies of inflammatory diseases including multiple sclerosis, infection and colitis. Here, we identified a novel small molecular compound Salt-inducible Kinases (SIKs) inhibitor HG-9-91-01 inhibiting necroptosis by targeting RIPK3 kinase activity. We found that SIKs inhibitor HG-9-91-01 could block TNF- or Toll-like receptors (TLRs)-mediated necroptosis independent of SIKs. We revealed that HG-9-91-01 dramatically decreased cellular activation of RIPK3 and MLKL. Meanwhile, HG-9-91-01 inhibited the association of RIPK3 with MLKL and oligomerization of downstream MLKL. Interestingly, we found that HG-9-91-01 also trigger RIPK3-RIPK1-caspase 1-caspase 8-dependent apoptosis, which activated cleavage of GSDME leading to its dependent pyroptosis. Mechanistic studies revealed that SIKs inhibitor HG-9-91-01 directly inhibited RIPK3 kinase activity to block necroptosis and interacted with RIPK3 and recruited RIPK1 to activate caspases leading to cleave GSDME. Importantly, mice pretreated with HG-9-91-01 showed resistance to TNF-induced systemic inflammatory response syndrome. Consistently, HG-9-91-01 treatment protected mice against *Staphylococcus aureus*-mediated lung damage through targeting RIPK3 kinase activity. Overall, our results revealed that SIKs inhibitor HG-9-91-01 is a novel inhibitor of RIPK3 kinase and a potential therapeutic target for the treatment of necroptosis-mediated inflammatory diseases.

## Introduction

Necroptosis is a regulatory form of cell death, independent of caspases activity, with cell membrane rupturing and release of damage associated molecular patterns (DAMPs) that trigger a strong innate immune response [1]. This caspase-independent cell death can be induced by specific death receptor, Interferon receptor, Toll-like receptor (TLRs), and ZBP-1 if the activity of caspase 8 is compromised [2]. Over the past few years, the molecular mechanism of necroptosis has been partially characterized. Upon the stimulation of TNF or FasL, RIPK1 and RIPK3 form a protein complex called necrosome through their RIP homotypic interaction motif (RHIM), which leads to the phosphorylation and oligomerization of RIPK3 [3–5]. However, For TLRs or DNA-dependent activator of interferon regulatory factors (DAI) induced necroptosis, downstream TRIF or DAI could replace RIPK1 to directly bind with RIPK3 leading to RIPK3 oligomerization and auto-phosphorylation [6–8]. Activated RIPK3 subsequently phosphorylates the downstream pseudokinase MLKL. Ultimately, phosphorylated MLKL undergoes oligomerization, translocates to cell membrane, interacts with phosphatidylinositides and results in cell membrane rupture [9–11]. In addition, increasing evidences also show that necroptosis perform a critical role in host defense against viral, bacterial and fungal infection [1, 12, 13]. Therefore, chemical inhibitors targeting necroptosis signaling pathway are therapeutic potential for necroptosis-associated diseases.

The necroptosis signaling pathways are stringently regulated, especially in the process of embryonic development [13]. Depletion of apoptosis-associated proteins including Caspase 8, FADD and cFLIP lead to embryonic lethality, which can be rescued by ablation of RIPK3 or MLKL [14, 15]. Consistently, mice with conditional deletion of caspase 8 or FADD in intestinal epithelium developed spontaneously colitis that was dependent on MLKL and RIPK3-mediated epithelial cell necroptosis [15, 16]. On the other hand, mice deficient with regulators such as CHIP and A20 similarly exhibit dwarfism and postnatal lethality, which also can be partially rescued by RIPK3 knock out [17, 18]. Therefore, necroptosis is sophisticatedly regulated under physiological conditions. Other studies showed that kinases including IKKα, IKKβ, TBK1, and MK2 directly targeted and phosphorylated RIPK1 and restricted the auto-phosphorylation of RIPK1, which restrained the activation of TNF induced apoptosis and necroptosis [19–21]. Besides RIPK1, RIPK3 is also tightly regulated. Casein kinase-1γ1 and 3 can be recruited to the necrosome complex and directly phosphorylate RIPK3 to promote TNF-induced necroptosis [22]. Additionally, it was reported that ppm1b inhibited the recruitment of MLKL to necrosome complex by dephosphorylating RIPK3 [23]. Heat shock protein 90 and cochaperone CDC37 complex have been shown to promote RIPK3 activation [24]. Lots of studies showed that RIPK1 and RIPK3 were differently controlled by kinase or enzymes [25]. Recently, a study revealed that TAM Kinases (Tyro3, Axl, and Mer) phosphorylated MLKL at the Tyr376 site to promote its oligomerization [26].

Previous work clarified that GSK’872, GSK840, and GSK843 inhibited TNF, TLRs or DAI-induced necroptosis by interacting with RIPK3 to trigger caspase 8 dependent apoptosis at high concentrations [27]. Although mice expressing RIPK3 D161N mutant faced embryonic lethal due to RIPK1- and caspase-8-dependent apoptosis, mice hold RIPK3 K51A was viable and fertile [27, 28]. Therefore, RIPK3 kinase activity is not the crucial factor that determines whether a cell activates caspase-8 and dies by apoptosis. There are other small molecular inhibitors that target RIPK3 kinase activity and alleviate TNF-induced systemic inflammatory response syndrome [29]. Some studies find that FDA-approved drugs sorafenib, dabrafenib and ponatinib inhibit RIPK3 kinase activity though as off-targets effect [29].

In the current study, we found that SIKs inhibitor HG-9-91-01 blocked TNF- and TLRs-induced necroptosis, which appeared to be independent of HG-9-91-01-mediated inhibition of SIKs. SIKs inhibitor HG-9-91-01 inhibited RIPK3 auto-phosphorylation, downstream phosphorylation and oligomerization of MLKL. Consistently, like molecular inhibitor GSK’872, HG-9-91-01 also induced RIPK1-RIPK3-Caspase 1-Caspase 8-dependent apoptosis, which cleaved downstream GSDME to trigger pyroptosis. Further investigation revealed that the function of HG-9-91-01 was mediated by directly inhibiting RIPK3 kinase activity with high efficiency. Importantly, treatment with HG-9-91-01 protects mice from TNF-induced systemic inflammatory response syndrome (SIRS) and *Staphylococcus aureus*-mediated lung damage.

## Materials and methods

### Antibody and reagent

zVAD, HG-9-91-01, and YKL were purchased from MedchemExpress. Necrostatin-1 was from Calbiochem. GSK’872 and SM-164 were from Selleck. Protein A/G Magnetic Beads were purchased from Pierce. Lipofectamine 2000 was from Life Technologies. LPS and poly I:C were purchased from InvivoGen. Mouse and human recombinant TNF were purchased from Novoprotein. Propidium iodide (PI) and cycloheximide (CHX) was from Sigma. Human recombinant RIPK1 and RIPK3 were from AtaGenix. Elisa kits for TNF and IL-1β were purchased from R&D. ADP-Glo kinase assay kit was from Promega. Immunoblotting was performed with following antibody: anti-RIPK1 (610459, BD Biosciences); anti-hRIPK1 S166 (44590, Cell Signaling); anti-mRIPK1 S166 (31122, Cell Signaling); anti-hRIPK3 (13526, Cell Signaling); anti-mRIPK3 (2283, ProSci); anti-hRIPK3 S227 (91702, Cell Signaling); anti-mRIPK3 T231/S232 (91702, Cell Signaling); anti-hMLKL (ab184718, Abcam); anti-mMLKL (AP14272b, ABGENT); anti-hMLKL S358 (ab187091, Abcam); anti-mMLKL S345 (ab196436, Abcam); anti-GAPDH (sc-32233, Santa Cruz); anti-cleaved Caspase-8 Asp387 (8592, Cell Signaling); anti-cleaved Caspase-3 (9661, Cell Signaling); anti-GSDME (ab215191, Abcam), anti-GSDMD (ab209845, Abcam), anti-Caspase-1 (AG-20B-0042-C100, AdipoGen), anti-Caspase-11 (NB120-10454, Novus).

### Cell culture

293 T cells were cultured in completed Dulbecco’s modified Eagle’s medium supplemented with 10% FBS, 100 U/mL penicillin and 100 μg/mL streptomycin. L929 and HT-29 cells were cultured in completed 1640 medium supplemented with 10% FBS, 100 U/ml penicillin and 100 μg/ml streptomycin. Peritoneal macrophages were isolated and cultured as described previously [30]. Briefly, wild-type mice were injected intraperitoneally with 2 ml 4% thioglycolate medium (FTG from BD Biosciences). In total, 3 days later, Cells were harvested by peritoneal cavity lavage with sterile PBS and seeded in the plates in DMEM complete medium. After 3 h, the supernatant cells were discarded and the adherent cells were regarded as peritoneal macrophages.

### Mice

Wild-type C57BL/6 mice were purchased from Gempharmatech. All mice were maintained in specific-pathogen-free conditions at the Shenzhen-Peking University-the Hong Kong University of Science and Technology Medical Center. 6 to 8 weeks old and gender matched mice were used in all experiments. All animal experiments were performed according to the guidelines for the care and use of laboratory animals and are approved by the institutional biomedical research ethics committee of the Guangdong Medical University.

### Cell death and survival assays

Cell death was determined by measuring the levels of released cytoplasmic enzyme lactate dehydrogenase (LDH) or staining with propidium iodide (PI). For the LDH release assay, cells were seeded in 96 or 24 well plates. Before treatment, media was changed with OPTI MEM. Cells were treated as indicated and the supernatant was collected to detect the released LDH using the CytoTox96 LDH release kit (Promega), according to the manufacture’s instruction. Total LDH was produced by adding lysis buffer to liberate all cytoplasmic LDH. Cell viability was calculated with percentage of total LDH. For the PI staining, Cells were treated with indicated stimulus and then PI (5 μg/mL) was directly added to medium and incubated for 20 min. The pictures were produced with an inverted fluorescence microscope.

### Generation of *Ripk3* knockout L929 cells by CRISPR-Cas9

Lentivirus was packaged in HEK293T cells transfected with *Ripk3* sgRNA (GAGTTAATGATTCATTGCTG)-expressing lentiCRISPRv2 puro together with dR8.2 and VSVG. The supernatant was collected for 72 h and filtered for subsequent L929 cells infection. Three days after infection, infected cells were selected with 4 mg/ml puromycin for 48 h and diluted to single clones cultured in 96-well plates. The *Ripk3* knockout clones were confirmed by sequencing of genomic DNA and immunoblot.

### Real-time PCR

RNA was obtained from cells using Trizol according to the manufacturer’s protocol. cDNA was produced by retro-transcribing RNA using PrimeScript RT reagent kit (Takara). Real-time PCR was performed using SYBR Premix ExTaq kit (TaKaRa) on ABI PRISM 7500 Sequence Detection System (Applied Biosystems), according to the manufacturer’s instructions. The gene expression results were normalized to the housekeeping gene *Rpl13a*. Real-time PCR primers were shown in Supplementary Table 1.

### Immunoprecipitation and immunoblotting

Immunoprecipitation and immunoblot analysis were performed as described previously [30]. Briefly, the cells were treated as indicated and washed with cold PBS. The cells were harvested with lysis buffer (50 mM Tris-HCl (pH 7.5), 150 mM NaCl, 1.0% Triton X-100, 1 mM EDTA and 10% glycerol containing protease inhibitors). Cell extracts were left on ice for 30 min and centrifuged as 13000 r.p.m for 30 min. Cell lysates were incubated with 2 μg anti-RIPK3 antibody or anti-Flag antibody overnight at 4 °C and then incubated with 15 μl protein A/G Magnetic beads for additional 3 h at 4 °C. Beads were washed with lysis buffer for three times and then boiled in 20 μL 3 × SDS loading buffer for 10 min. Samples were loaded on SDS-PAGE gels followed by electroblotting onto PVDF membranes (Millipore). To determine protein levels, immunoblotting was performed using the indicated antibodies.

### RNA interference

L929 cells and peritoneal macrophages were seeded in 96-well plates to detect cell viability or in 24-well plate to harvest protein to detect knockdown efficiency. Cells were transiently transfected with indicated siRNA (Scrambled siRNA (Negative control)), RIPK3 siRNA (5′-gcucucgucuucaacaacu-3′), MLKL siRNA (5′-gaaccugcccgaugacauu-3′), CRTC3 siRNA (5′-guuucgagcugaccggcua-3′), RIPK1 siRNA (5′-gcucucgucuucaacaacu-3′), Caspase 8 siRNA (5′-gugaauggaaccugguaua-3′), SIK1 siRNA (5′-aaacgcagguugcaauaaa-3′), SIK2 siRNA (5′-gcgucggccuagcaccauu-3′), SIK3 siRNA (5′-ccaaauaagcgccucucaa-3′), GSDME siRNA (5′- gcugcaaacuccauguuau-3′), GSDMD siRNA (5′-caacugcuuauuggcucuaaa-3′), Caspase 1 siRNA (5′-gacauuaaacgaagaauccaguuca-3′), Caspase 11 siRNA (5′-gugcaacaaucauuugaaa-3′) according to manufacturer’s protocol using Lipofectamine RNAiMAX (Life Technologies). Cells were incubated with transfectin mixture for 3 days, and then stimulated with indicated ligands and compounds. All of siRNA were purchased from Genepharma.

### TNF-induced SIRS model

6–8 week old female mice (*n* = 5–8/group) were used for experiments. Mice were injected intraperitoneally (i.p.) with DMSO or HG (5 mg/kg) for 30 min and then injected intravenously with 5 μg per mice mouse recombinant TNF diluted in endotoxin free PBS. Mortality of mice was monitored every hour after TNF injection. Serum and tissue samples of uterus, liver and caecum were collected at indicated times after injection.

### Staphylococcus aureus infection

*Staphylococcus aureus* USA300 was from ATCC. In total, 6–8 weeks old C57BL/6 female mice (*n* = 5/group) were injected intraperitoneally with DMSO or HG (5 mg/kg) for 30 min. And then mice were intranasally infected with 10^7^ Colony forming units (CFU) per mouse *Staphylococcus aureus*. In total, 24 h later, bronchoalveolar lavage fluids were collected by instilling 1 ml PBS into the trachea of euthanized mice before lungs were removed. Then, lungs from infected mice were harvested for histology and measuring CFU. Homogenized lungs were diluted with sterile PBS and plated on Luria Bertani (LB) agar plates. CFU was counted after 12 h on LB agar plates at 37 °C.

### Histology

After mice were euthanized, tissues were collected and fixed in 4% paraformaldehyde for at least 24 h. Fixed samples were embedded into paraffin and sliced into 5 μm sections. In total, 5 μm sections were stained with H&E, according to the standard procedures described previously. The images were captured using with a Leica FDM2500 microscope.

### RIPK3 and MLKL oligomerization

Peritoneal macrophages were seeded in 24-well plates and treated as indicated. Cells were washed with cold PBS and harvested with 2×DTT-free sample buffer (125 mM Tris-Cl PH 6.8, SDS 4%, Glycerol 20%, Bromophenol blue 0.02%). Samples were loaded on SDS-PAGE gels followed by electroblotting onto PVDF membranes (Millipore). Immunoblot analysis was performed with RIPK3 and MLKL antibody.

### In Vitro Kinase activity assay

Recombinant human RIPK1 and RIPK3 protein were incubated with indicated compounds for 30 min in the kinase reaction buffer (25 mM Herpes pH 7.2, 12.5 mM MnCl2, 20 mM Mgcl2, 5 mM EGTA, 2 mM EDTA, 12.5 mM b-glycerol phosphate, and 2 mM DTT). After that, ATP (50 μM) and substrate MBP (20 μM) were added into kinase reaction buffer for 2 h. Kinase activity was determined by detecting luminescence using ADP-Glo kinase assay kit (Promega) according to manufacturer’s protocol.

### Immunofluorescent staining

Peritoneal macrophages were seeded in chamber slide and cultured overnight. Cells were pretreated with HG for 1 h and then treated with TNF, LPS, poly I:C or zVAD for 12 h. The cells were washed with PBS and fixed in 4% paraformaldehyde for 15 min. Cells were washed three times and incubated in blocking buffer containing 5% goat serum and 0.3% Triton™ X-100 for 1 h. Later, cells were incubated with anti-RIPK1 S166 antibody, anti-RIPK3 T231/S232 antibody or anti-MLKL S345 antibody overnight at 4 °C. On the following day, cells were incubated with secondary antibody for 1 h. Nuclei were stained with DAPI for 5 min. Images were produced with Zeiss microscope.

### Statistical analysis

Software Prism 8 (GraphPad Software) was used to perform statistical analysis and graph development. Two-tailed Student’s *t* test was used to compare differences between two groups. Survival curves were presented using Kaplan-Meyer method and significance was calculated by log-rank (Mantel-Cox) test. Data are shown as the mean ± standard error of the mean (SEM). All experiments were performed at least three times. Non-cropped western blots data were shown in supplementary materials. Statistical significance was defined as *P* < 0.05. *P < 0.05, **P < 0.01, ***P < 0.001.

## Results

### SIKs inhibitor HG-9-91-01 dramatically blocks TNF- and TLRs-induced necroptosis

**Fig. 1:**
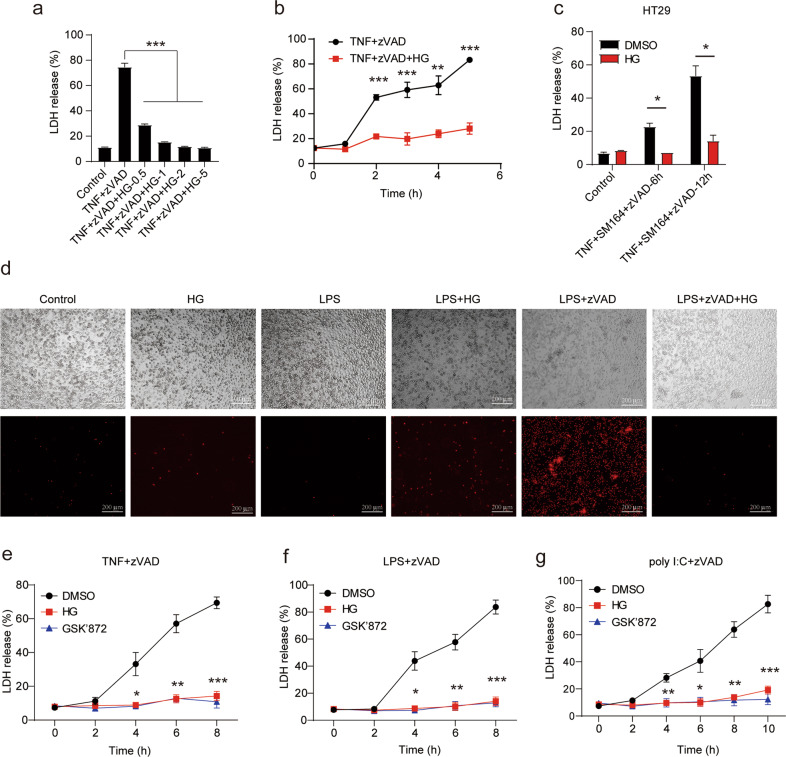
SIKs inhibitor HG-9-91-01 blocked TNF- and TLR-induced necroptosis in human and mouse cells. **a** L929 cell were pretreated with indicated concentrations of HG-9-9-01 (HG) for 30 min prior to treatment with mouse TNF (10 ng/mL) and zVAD (20 μM) for 12 h. Cell death was determined by released lactate dehydrogenase (LDH). Bars represent the mean ± SEM from at least three independent experiments performed in triplicate. **b** L929 cell were pretreated with HG (5 μM) for 30 min and then treated with mouse TNF and zVAD for indicated time. Cell death was determined by released LDH. Bars represent the mean ± SEM from at least three independent experiments performed in triplicate. (**c**) HT-29 cell were pretreated with HG (5 μM) for 30 min followed with treatment with human TNF (30 ng/mL), SM-164 (1 μM), zVAD for indicated time. Cell death was determined by released LDH. Bars represent the mean ± SEM from at least three independent experiments performed in triplicate. **d** Peritoneal macrophages from wild type mice were pretreated with (1 μM) HG for 30 min, followed by treatment with LPS (25 ng/mL) and zVAD for 12 h. Cells were stained with propidium iodide (PI). Photomicrographs of histology are shown at ×100 magnification. Results are representative of at least three independent experiments. **e-g** Peritoneal macrophages were pretreated with (1 μM) HG for 30 min followed by treatment with LPS, zVAD, poly I:C (30 μM), or TNF (50 ng/mL) for indicated time. Cell death was determined by released lactate dehydrogenase (LDH). Bars represent the mean ± SEM from at least three independent experiments performed in triplicate. Student’s *t* test, *p < 0.05, **p < 0.01, ***p < 0.001.

Previous work showed that IKKi and TBK1 phosphorylated RIPK1 to inhibit TNF-induced apoptosis and RIPK1 kinase mutant mice rescued embryonic lethality of TBK1 deficiency mice [21]. We also found that small molecular inhibitor MRT 67307 targeting IKKi and TBK1 kinase activity promoted TNF- and TLRs-induced necroptosis before the published work (Supplementary Fig. 1a, b). However, inhibitor MRT 67307 still functioned to promote TNF- and TLR-induced necroptosis in IKKi deficient macrophages transfected siRNA targeting TBK1 (data not shown). Because of that MRT 67307 also targets ULK1, ULK2 and SIKs (SIK1, SIK2, and SIK3). Therefore, we employed ULK-101 targeting ULK1 and ULK2, HG-9-91-01 targeting SIKs, and surprisingly found that HG-9-91-01 efficiently inhibit TNF- and TLRs-induced necroptosis and ULK-101 had no significant effect on necroptosis (Supplementary Fig. 1c). To investigate the roles of SIKs inhibitor HG-9-91-01 in necroptosis, we pretreated L929 cells with HG-9-91-01 and triggered necroptosis with TNF plus zVAD. Treatment with HG-9-91-01 exhibited efficient inhibition of TNF-induced necroptosis in L929 cells in a concentration-dependent manner (Fig. 1a). In addition, HG-9-91-01 could inhibit TNF-induced necroptosis in an early time point (Fig. 1b). In both HT-29 cells and peritoneal macrophages, HG-9-91-01 indeed blocked the TNF-triggered necroptosis (Fig. 1c, e and Supplementary Fig. 4b, c). We also checked the effect of HG-9-91-01 on TLR4-induced necroptosis triggered by LPS plus zVAD and TLR3-induced necroptosis initiated by poly I:C plus zVAD. Consistently, HG-9-91-01 also blocked TLRs-induced necroptosis in macrophages (Fig. 1f, g). To confirm the inhibitory effect of HG-9-91-01, we performed a PI staining assay in peritoneal macrophages. Our results revealed that macrophages pretreated with HG-9-91-01 showed less PI-positive staining compared with control macrophages pretreated with DMSO (Fig. 1d, Supplementary Fig. 1d, e). Overall, these data indicate that SIKs inhibitor HG-9-91-01 efficiently block TNF- and TLR-induced necroptosis.

### SIKs are not required for TNF- and TLRs-induced necroptosis

**Fig. 2:**
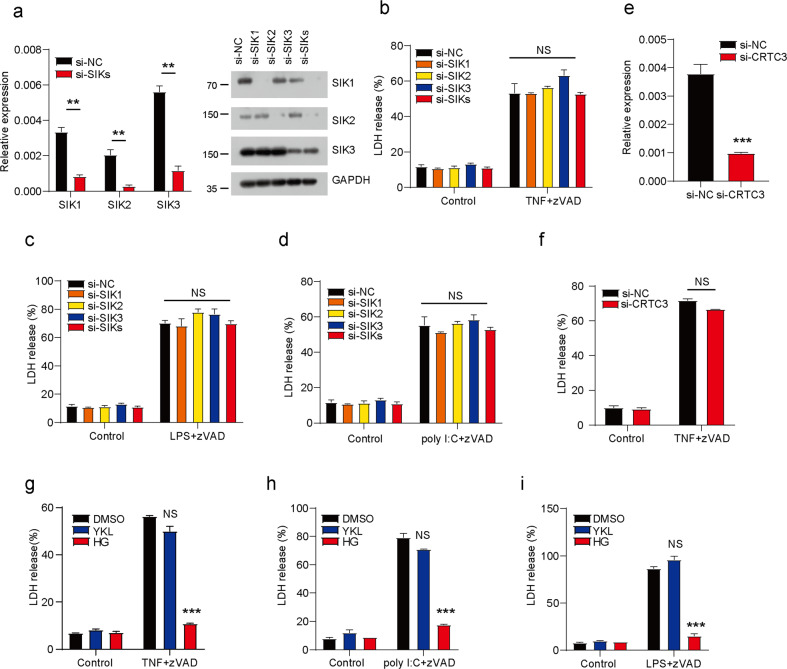
SIKs were not the targets of HG-9-91-01 for blockage of TNF- and TLR-induced necroptosis. **a** Peritoneal macrophages were transfected with indicated siRNA for 3 days. Knockdown efficiency was determined by RT-PCR. Bars represent the mean ± SEM from at least three independent experiments. Peritoneal macrophages were transfected with indicated siRNA for 3 days and then treated with TNF + zVAD (**b**), LPS + zVAD (**c**), or poly I:C + zVAD (**d**) for 12 h. Cell death was determined by released LDH. Bars represent the mean ± SEM from at least three independent experiments performed in duplicate. **e**, **f** Peritoneal macrophages were transfected with specific siRNA targeting CRTC3 for 3 days and then treated with TNF + zVAD for 12 h. **e** Knockdown efficiency was determined by RT-PCR. **f** Cell death was determined by released LDH. Bars represent the mean ± SEM from at least three independent experiments performed in duplicate. Peritoneal macrophages were pretreated with YKL or HG for 30 min and then treated with TNF (**g**), LPS (**h**), poly I:C (**i**) or zVAD for 12 h. Cell death was determined by released LDH. Bars represent the mean ± SEM from at least three independent experiments performed in triplicate. Student’s *t* test, *p < 0.05, **p < 0.01, ***p < 0.001.

It is known that SIKs (SIK1, SIK2, and SIK3) are the functional targets of HG-9-91-01 [31]. Our above data showed that HG-9-91-01 efficiently inhibited necroptosis. Therefore, we speculated that SIKs might be required for necroptosis. We manipulated macrophages by transfecting with indicated siRNA to knockdown the SIK1, SIK2 and SIK3 mRNA levels (Fig. 2a). However, macrophages with single deficiency or treble deficiencies of SIKs showed comparable necroptosis triggered by TNF or TLRs compared with control macrophages (Fig. 2b–d). We knocked down CRTC3 which was a downstream factor of SIKs in L929 cells (Fig. 2e), which played various roles in cancer and inflammation [32]. Our data showed that CRTC3 was dispensable for TNF-induced necroptosis (Fig. 2f). On the other hand, we also employed another SIKs inhibitor YKL-06-061 that had been shown high efficient inhibition of SIKs [33]. However, macrophages pretreated with YKL-06-061 showed similar necroptosis induced by TNF or TLRs (Fig. 2g-i). Collectively, our results demonstrate that SIKs are not required for TNF- and TLRs-induced necroptosis, and the inhibition of necroptosis mediated by HG-9-91-01 is an off-target effect.

### HG-9-91-01 decreases downstream signaling activation of RIPK3 and MLKL in TNF- and TLRs-induced necroptosis

**Fig. 3:**
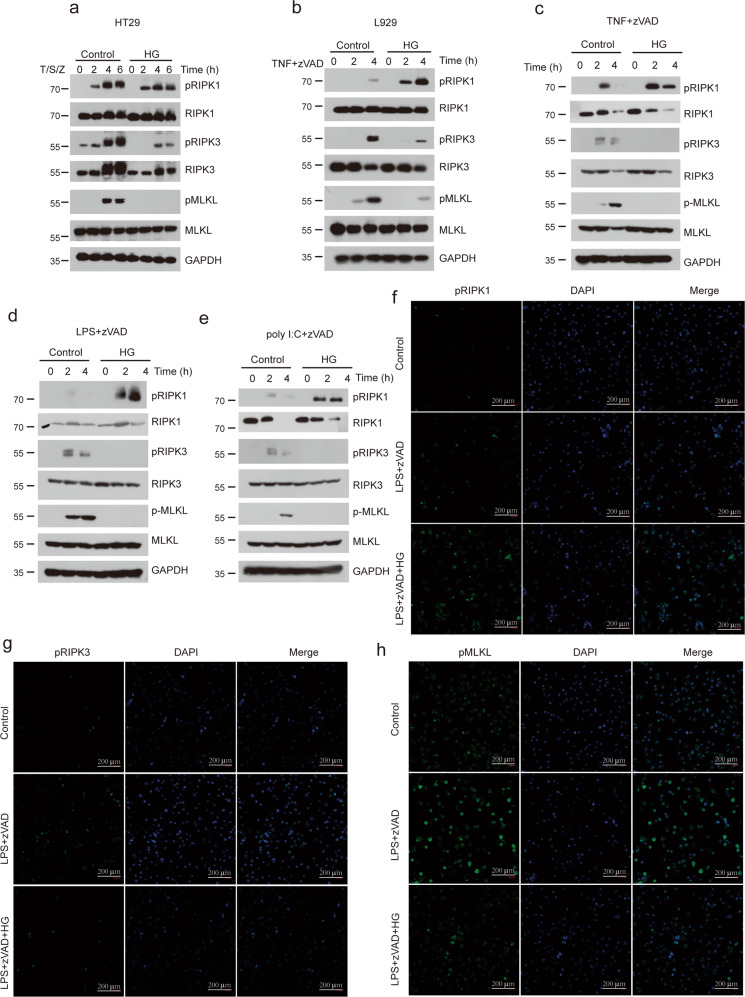
HG-9-9-01 decreased activation of RIPK3 and MLKL. **a** HT-29 cell were pretreated with HG for 30 min followed by treatment with TNF, SM-164, and zVAD for indicated time. Phosphorylation of RIPK1, RIPK3, and MLKL were assessed by immunoblotting with the indicated antibodies. **b** L929 cell were pretreated with HG for 30 min followed by treatment with TNF and zVAD for indicated time. Phosphorylation of RIPK1, RIPK3 and MLKL were assessed by immunoblotting with the indicated antibodies. Peritoneal macrophages were pretreated wit HG for 30 min and then treated with TNF + zVAD (**c**), LPS + zVAD (**d**), or poly I:C + zVAD (**e**) for indicated time. Phosphorylation of RIPK1, RIPK3 and MLKL were assessed by immunoblotting with the indicated antibodies. **f**–**h** Peritoneal macrophages were pretreated with HG for 30 min and then treated with LPS + zVAD for 3 h. Phosphorylation of RIPK1 S166 (**f**), RIPK3 (**g**), and MLKL (**h**) were assessed by immunofluorescence. Results are representative of at least three independent experiments.

To investigate the mechanism underlying HG-9-91-01-mediated inhibition of necroptosis, we checked the downstream NF-κB signaling of TNF. We found that cells pretreated with HG-9-91-01 had comparable activation of NF-κB signaling (Supplementary Fig. 2a). Gene induction of chemokine seemed also to be similar (Supplementary Fig. 2b, c). So we could exclude the possibility that HG-9-91-01 blocked TNF-induced necroptosis by regulating TNF-induced NF-κB activation. Therefore, we checked the phosphorylation of RIPK1, RIPK3, and MLKL. Our results showed that treatment of HG-9-91-01 abolished phosphorylation of RIPK3 and MLKL in HT29 cells (Fig. 3a). Similar results were observed in mouse cell lines L929 treated with TNF plus zVAD (Fig. 3b). Although HG-9-91-01 had no significant effect on RIPK1 auto-phosphorylation in human cells (Fig. 3a), it promoted the RIPK1 auto-phosphorylation in mouse cells (Fig. 3b). To confirm the effect of HG-9-91-01 on the necroptotic signaling pathway, we checked the activation of RIPK1, RIPK3 and MLKL. Similarly, HG-9-91-01 could increase RIPK1 auto-phosphorylation and abolish phosphorylation of RIPK3 and MLKL (Fig. 3c–e). Results from immunofluorescence staining of phosphorylated RIPK1, RIPK3 and MLKL induced by TNF or TLRs were also concordant with the immunoblot data (Fig. 3f–h). These results provide strong evidence that HG-9-91-01 blocks TNF- or TLRs-induced necroptosis by abolishing RIPK3 and MLKL activation.

### HG-9-91-01 disrupts the association between RIPK3 and MLKL

**Fig. 4:**
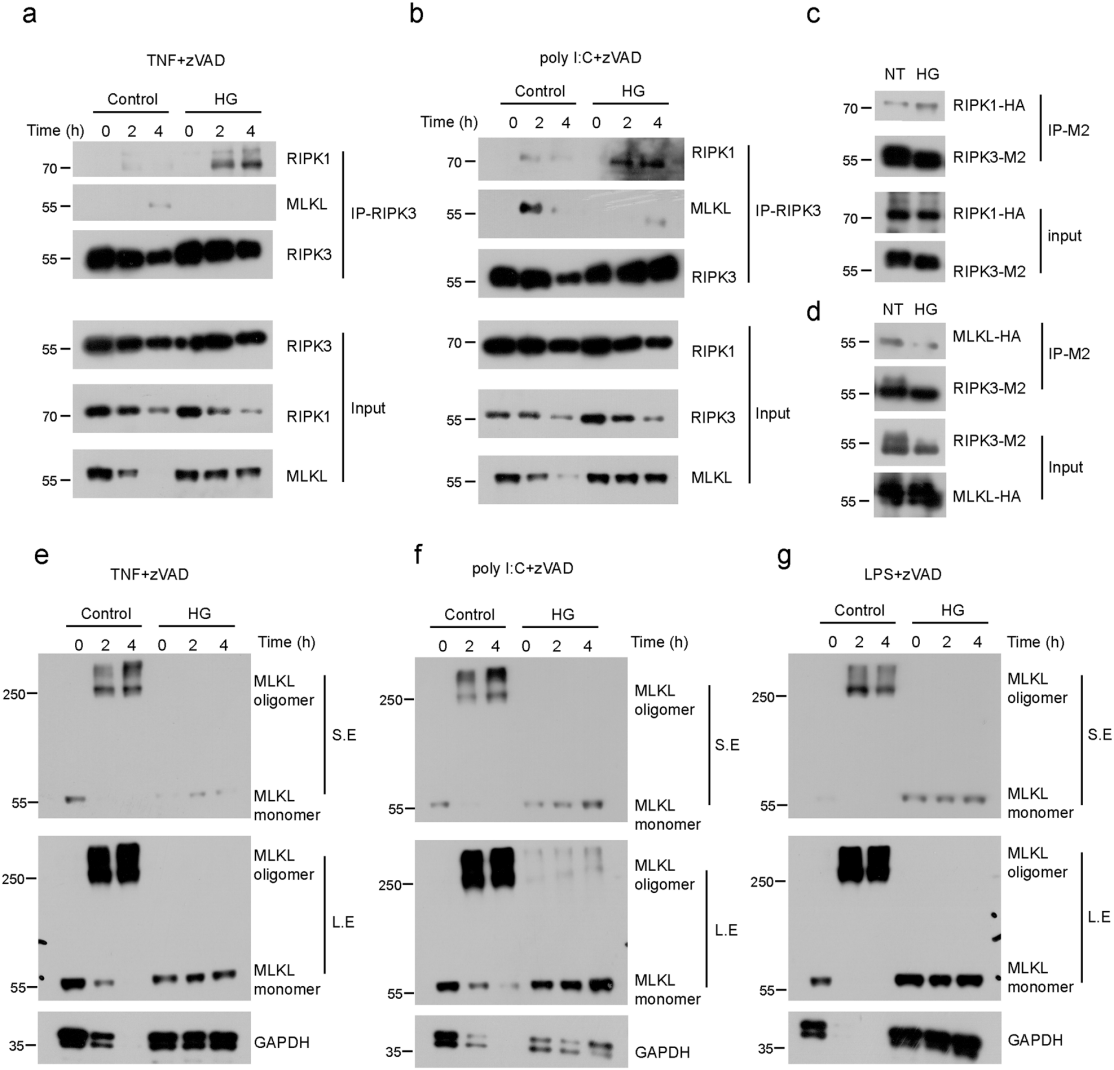
HG-9-9-01 inhibits interaction of RIPK3 and MLKL and downstream signaling cascade. Peritoneal macrophages were pretreated with HG for 30 min and the treated with TNF + zVAD (**a**) and poly I:C + zVAD (**b**) for indicated periods of time. Total cell lysates were employed for immunoprecipitation using anti-RIPK3 antibody. Cell lysates were immunoblotted with the indicated antibody. 293 T cells were ectopically expressed plasmid of RIPK3-M2 and RIPK1-HA (**c**), RIPK3-M2 and MLKL-HA (**d**) for 24 h and then treated with HG for 3 h. Cell lysates were immunoprecipitated using anti-Flag antibody and immunobloted with the indicated antibody. Peritoneal macrophages were pretreated with HG for 30 min followed by treatment with TNF + zVAD (**e**), LPS + zVAD (**f**), or poly I:C + zVAD (**g**) for indicated time. MLKL oligomerization was determined by Non-reducing PAGE. Results are representative of at least three independent experiments.

The formation of RIPK1 and RIPK3 necrosome complex has been a key step for TNF-induced necroptosis [2]. We performed immunoprecipitation experiment to analyze the interaction of RIPK3 and RIPK1 or RIPK3 and MLKL. In the presence of HG-9-91-01, RIPK3 could recruit more RIPK1 to necrosome complex induced by TNF-mediated necroptosis (Fig. 4a). However, peritoneal macrophages treated with HG-9-91-01 had a dis-association of RIPK3 with MLKL (Fig. 4a). The same attenuated association was observed in TLRs-induced necroptosis (Fig. 4b). These data implied that HG-9-91-01 inhibited TNF- or TLR-induced necroptosis by disrupting the association between RIPK3 and MLKL. To prove the hypothesis, we conducted co-immunoprecipitation analysis using overexpressed RIPK1, RIPK3 and MLKL in 293 T cells and found that HG-9-91-01 promoted interaction between RIPK3 and RIPK1 but decreased association of RIPK3 and MLKL, which was in agreement with the result from macrophages (Fig. 4c, d). Necroptosis can also trigger the oligomer formation of MLKL, which is considered a hallmark of necroptosis. Thus, we investigated whether oligomerization of MLKL was controlled by HG-9-91-01. Indeed, significant oligomerization of MLKL was observed in macrophages stimulated with TNF or TLRs in the presence of zVAD (Fig. 4e–g). However, treatment with HG-9-91-01 made the oligomerization of MLKL disappear (Fig. 4e-g and Supplementary Fig. 3a). Together, these results suggest that HG-9-91-01 destroys the interaction of RIPK3 and MLKL and abolishes the oligomerization of MLKL.

### HG-9-91-01 directly targets RIPK3 kinase activity

**Fig. 5:**
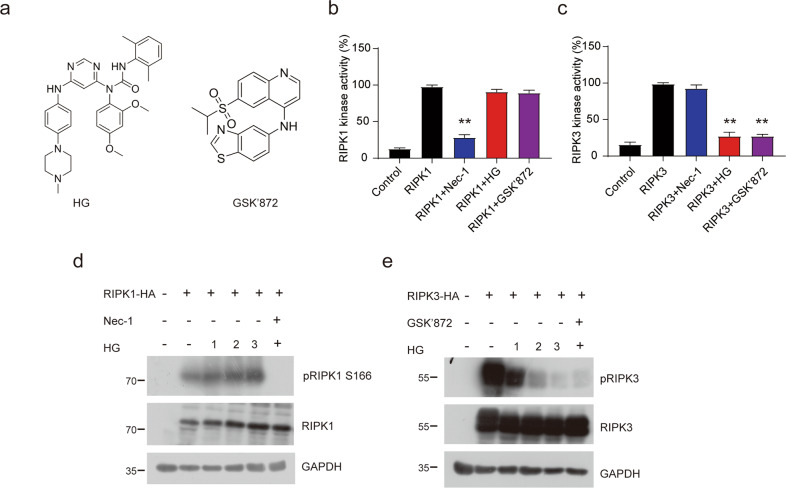
HG-9-9-01 directly targets RIPK3 kinase activity. **a** Chemical structure of HG-9-9-01 and GSK’872. **b** Response of the indicated small molecular compounds (20 μM Nec-1, 5 μM HG, and 10 μM GSK’872) on RIPK1 kinase activity of recombinant human RIPK1 assessed by ADP-Glo assay. Bars represent the mean ± SEM from at least three independent experiments. **c** Response of the indicated small molecular compounds on RIPK3 kinase activity of recombinant human RIPK3 assessed by ADP-Glo assay. Bars represent the mean ± SEM from at least three independent experiments. 293 T cells were overexpressed of RIPK1-HA (**d**) or RIPK3-HA (**e**) and treated with indicated dose HG for 3 h. Phosphorylation of RIPK1 or RIPK3 was measured by immunoblotting.

To explore the molecular mechanism underlying HG-9-91-01-mediated necroptosis suppression, we carry out in vitro kinase activity assays to analyze the inhibitory effect of HG-9-91-01. As previous reported [27, 34], Nec-1 could suppress the kinase activity of RIPK1 and GSK’872 failed to target RIPK1 kinase activity (Fig. 5b). Like GSK’872, HG-9-91-01 did not affect RIPK1 kinase activity (Fig. 5b). We subsequently checked the RIPK3 kinase activity and observed that GSK’872 indeed restrain RIPK3 kinase activity. Consistent with GSK’872, HG-9-91-01 also exhibited high inhibition of human RIPK3 kinase activity (Fig. 5c). Since ectopic overexpressing RIPK1 or RIPK3 promotes its auto-phosphorylation, which represents its kinase activity and can be detected with indicated antibody, we validate this phenomenon to verify HG-9-91-01-mediated inhibition of RIPK3. Our results revealed that RIPK1 auto-phosphorylation by overexpressing RIPK1 was not affected by HG-9-91-01 while the auto-phosphorylation was inhibited by Nec-1 (Fig. 5d). RIPK3 auto-phosphorylation by overexpressing RIPK3 was inhibited by HG-9-91-01 in a concentration-dependent manner (Fig. 5e). Collectively, these findings indicate that RIPK3 kinase activity is the direct target of HG-9-91-01.

### HG-9-91-01 triggers RIPK1-RIPK3-caspase 1-caspase 8 dependent cleavage of GSDME

**Fig. 6:**
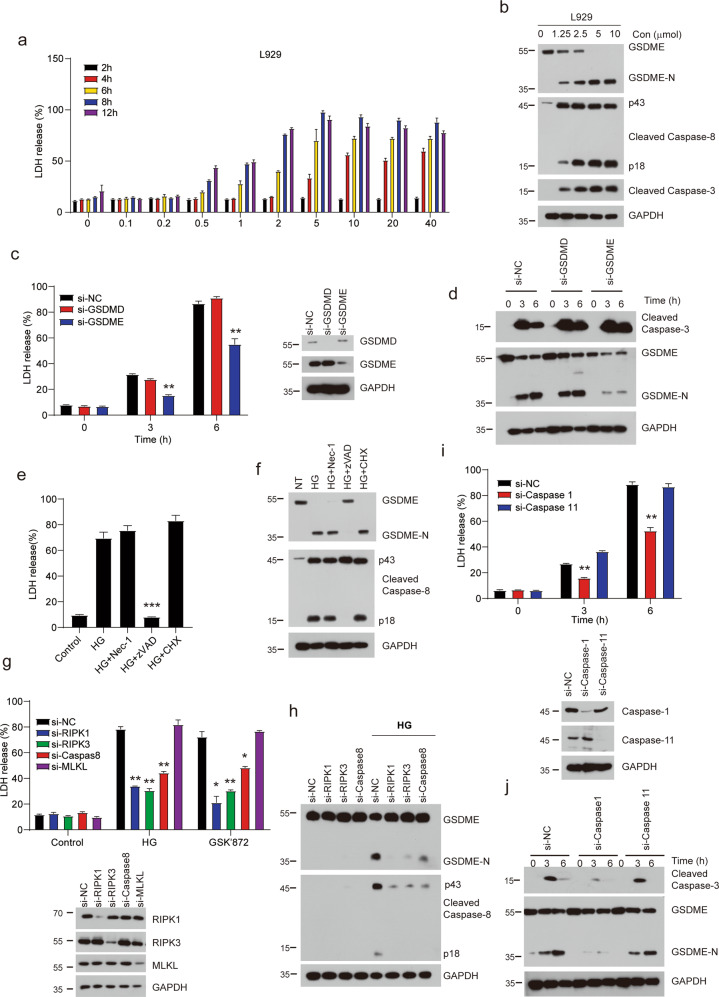
HG-9-9-01 triggered RIPK1-RIPK3-Caspase 1-Caspase 8-GSDME dependent pyroptosis. **a** Relative viability of L929 cells treated with increasing concentrations of HG for indicated time points was determined by LDH. Bars represent the mean ± SEM from at least three independent experiments. **b** L929 cells were treated with indicated concentrations of HG for 6 h. Cell lysates were analyzed with immunoblotting. **c**, **d** L929 cells were transfected with specific siRNA targeting *Gsdme* and *Gsdmd* for 3 days and then treated with 5 μM HG for indicated time. Cell death was determined by released LDH. Knockdown efficiency was represented on the right side. **c** Bars represent the mean ± SEM from at least three independent experiments. **d** Cell lysates were used with immunoblotting analysis. **e**, **f** L929 cells were pretreated with indicated compounds for 30 min followed by treatment of 5 μM HG for 6 h. **e** Cell death was determined by released LDH. Bars represent the mean ± SEM from at least three independent experiments. **f** Cell lysates from (**e**) were performed with immunoblotting for cleaved Caspase-8 and GSDME. **g** L929 cells were transfected with indicated siRNA for 3 days. Cells were then stimulated with 5 μM HG for 6 h. Cell death was determined by released LDH, and the below panel showed the knockdown efficiency Bars represent the mean ± SEM from at least three independent experiments. **h** L929 cells were transfected with indicated siRNA for 3 days. Cells were stimulated with 5 μM HG for 6 h. Cell lysates were harvested and immunoblotted with indicated antibodies. **i**, **j** L929 cells were transfected with specific siRNA targeting caspase 1 and caspase 11 for 3 days and then treated with 5 μM HG for indicated time. Cell death was determined by released LDH, the below panel showed the knockdown efficiency. **i** Bars represent the mean ± SEM from at least three independent experiments. **j** Cell lysates were used with immunoblotting analysis.

RIPK3 inhibitor GSK’872 has been reported not only to inhibit TNF- or TLRs-induced necroptosis but also to induce apoptosis at high concentrations [27]. Thus, we treated L929 cells with GSK’872 and observed that cell death was induced as previously reported [27] (Supplementary Fig. 4a). So we wonder whether HG-9-91-01 can trigger apoptosis. Similar results were obtained by treatment of HG-9-91-01, which is in time and dose dependent manner (Fig. 6a and Supplementary Fig. 5c, d). L929 cells showed more sensitive to HG-9-91-01 induced cell death. However, only high concentrations of HG-9-91-01 induced cell death in macrophages and MEFs. Caspase 3 and caspase 8 were activated by HG-9-91-01 (Fig. 6b). Recently, several works showed that GSDME had the function of switching caspases-mediated apoptosis activated by TNF or chemotherapy drugs to pyroptosis [35, 36]. Interestingly, we observed that HG-9-91-01-induced cell death was morphologically similar to those seen in pyroptosis. Cleaved GSDME accumulated under the treatment of HG-9-91-01 in a dose-dependent manner (Fig. 6b and Supplementary Fig. 5a, b, e). However, even high concentrations of HG-9-91-01 could not trigger significant cell death in THP1 and HT29 cells (Supplementary Fig. 5f, g). Depletion of GSDME made cells resistance to HG-9-91-01-induced cell death, which reinforced that HG-9-91-01 induced GSDME-dependent pyroptosis (Fig. 6c). However, knockdown of GSDMD showed no effect on HG-9-91-01-induced cell death and caspase 3 activation (Fig. 6c, d). We next investigated the pyroptosis induced by HG-9-91-01, and found that RIPK1 kinase inhibitor Nec-1 could not rescue cell death while pan-caspases inhibitor zVAD reversed cell death (Fig. 6e), indicating that RIPK1 kinase activity was dispensable for HG-9-91-01-mediated pyroptosis. Cleavage of GSDME and caspase 8 were also blocked by zVAD and were not disrupted by RIPK1 inhibitor Nec-1, which was concordant with the cell death (Fig. 6f). To investigate the properties of HG-9-91-01-mediated pyroptosis, we knocked down the levels of RIPK1, RIPK3, MLKL and caspase 8. Our data showed that cells deficient in RIPK1, RIPK3 or caspase 8 conferred resistances to HG-9-91-01-mediated pyroptosis (Fig. 6g). Knockout RIPK3 in L929 cells completely blocked HG-induced cell death, suggesting that HG-induced cell death was completely dependent on RIPK3 (Supplementary Fig. 5j). However, MLKL knockdown cells exhibited same sensitivity to HG-9-91-01-mediated pyroptosis (Fig. 6g). We also analyzed GSDME cleavage and found that it was dependent on RIPK1, RIPK3 and caspase 8 (Fig. 6h). It is well known that caspase 1 and caspase 11 are canonical caspases that cleaved gasdermins to execute pyroptosis. Therefore, we also knocked down the protein levels of caspase 1 and caspase 11 and found that depletion of caspase 1 made cell resistant to HG-9-91-01-mediated pyroptosis and cleavage of GSDME (Fig. 6i, j). Consistently, we also observed the same requirement of caspase-1 for GSK’872-induced cell death (Supplementary Fig. 5h, i). Thus, for HG-9-91-01-induced pyroptosis, RIPK1’s scaffold function not kinase activity, RIPK3, caspase 1 and caspase 8 are indispensable.

Besides HG-9-91-01-induced pyroptosis, we also found that HG-9-91-01 and GSK’872 promoted TNF-induced cell death at low concentration, which did not induce dramatic pyroptosis in L929 cells (Supplementary Fig. 4d). However, HG-9-91-01 plus TNF or TLRs did not induce significant cell death in macrophages (Supplementary Fig. 4e). Interestingly, HG-0-91-01 dramatically promotes TNF plus SM164-induced cell death in macrophages (Supplementary Fig. 4e). However, unlike HG-9-91-01-induced pyroptosis, knockdown of RIPK1 showed sensitive to TNF plus HG-9-91-01-induced cell death (Supplementary Fig. 4f). Deficient of RIPK3 or MLKL displayed resistance to TNF plus HG-9-91-01-induced cell death (Supplementary Fig. 4g, h). Pretreated with pan-caspase inhibitor zVAD blocked TNF plus HG-9-91-01-induced cleavage of GSDME and caspase 3 activation (Supplementary Fig. 4i). Overall, our data revealed that RIPK3 and MLKL are indispensable for TNF plus HG-9-91-01-induced pyroptosis, which is not the same as HG-9-91-01-induced pyroptosis.

### HG-9-91-01 attenuates necroptosis-associated inflammatory injury by targeting RIPK3 kinase activity

**Fig. 7:**
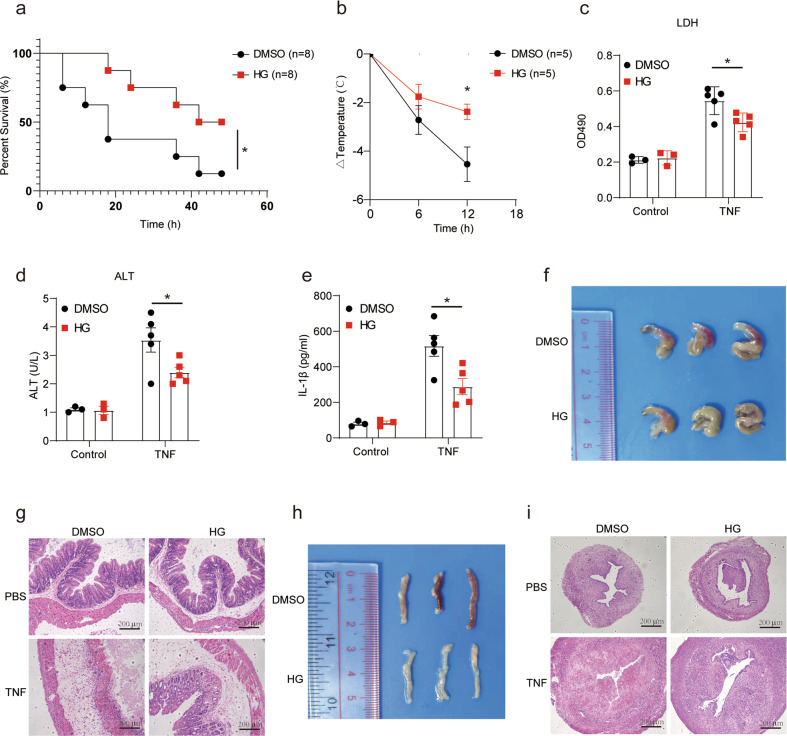
HG-9-9-01 alleviated mice from TNF-induced SIRS in vivo. **a**, **b** Wild-type female mice were injected i.p. with DMSO (*n* = 8) or HG (*n* = 8) for 30 min prior to a challenge with 5 μg mTNF per mice through the tail vein. Mouse survival (**a**) and body temperature (**b**) was monitored. **c**–**e** Plasma samples of mice treated with DMSO or HG were collected 12 h after challenge with TNF or PBS and analyzed for IL1β protein level (**e**) and activities of LDH (**c**) and ALT (**d**). **f** Macroscopic view of the representative caecums from mice treated as in (**a**) for 12 h. **g** H&E histology of the representative caecums from mice treated as in (**a**). Photomicrographs of histology are shown at ×100 magnification. **h** Macroscopic view of the representative uterus from mice treated as in (**a**) for 12 h. **i** H&E histology of the representative uterus from mice treated as in (**a**). Photomicrographs of histology are shown at ×100 magnification. Results are representative of at least three independent experiments. Error bars represent SEM. Survival curves were presented using Kaplan-Meyer method and significance was calculated by log-rank (Mantel-Cox) test, *p < 0.05.

According to the HG-9-91-01-mediated inhibition of RIPK3 kinase activity, we sought to investigate the therapeutic potential of HG-9-91-01 in necroptosis-associated inflammatory injury. TNF-induced systemic inflammatory response syndrome (SIRS) is the most used models for proving the roles in necroptosis as RIPK3 or MLKL KO mice are resistant [37]. Although our data implied that RIPK3 inhibitor (HG-9-91-01 or GSK’872) could induce apoptosis or promote TNF-induced apoptosis, apoptosis was not the determined process for TNF induced SIRS [37]. Mice treated with HG-9-91-01 were more resistant to TNF-induced SIRS than the control group, which were protected from death and severe hypothermia (Fig. 7a, b). Consistently, the releasing of plasma alanine aminotransferase (ALT) and LDH from damaged tissue was significantly lower in mice treated with HG-9-91-01 than in control mice (Fig. 7c, d). Cytokine IL-1β was significantly lower in mice treated with HG-9-91-01 (Fig. 7e). TNF-induced caecum damage was protected by HG-9-91-01 (Fig. 7f, g). Interestingly, similar to caecum, we found that the uterus was damaged in TNF-induced SIRS and HG-9-91-01 could alleviate TNF-induced uterus damage (Fig. 7h, i). Collectively, these results demonstrate that HG-9-91-01 can alleviate TNF-induced SIRS by targeting RIPK3 kinase activity.

**Fig. 8:**
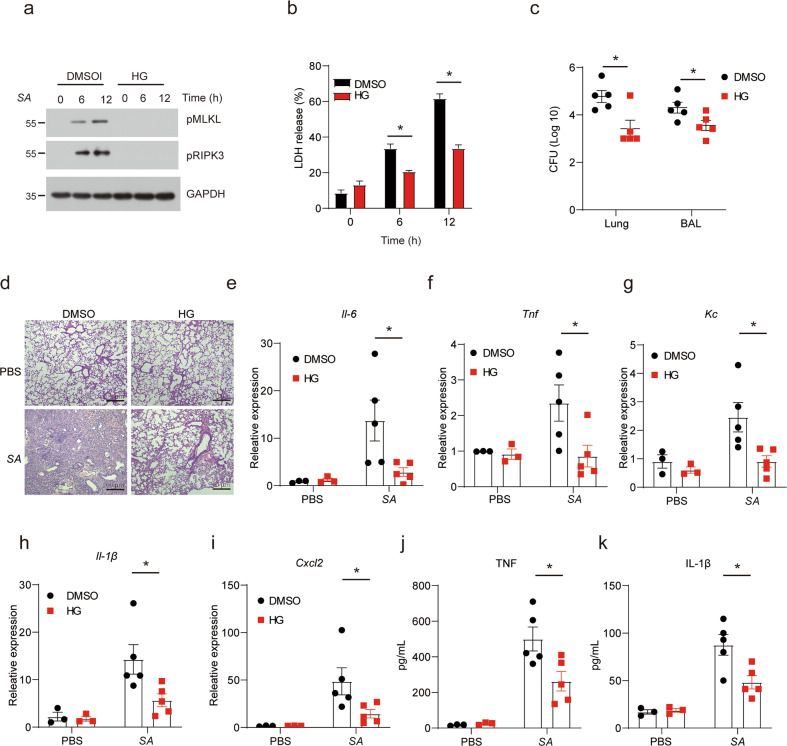
HG-9-9-01 relieved *Staphylococcus aureus*-mediated lung injury by targeting RIPK3 kinase activity. **a** Peritoneal macrophages were pretreated with DMSO or HG for 30 min and then treated with MOI 1 *Staphylococcus aureus* (SA) for the indicated time. Cell lysates were used for immunoblotting with indicated antibodies. **b** Peritoneal macrophages were pretreated with DMSO or HG for 30 min and then treated with MOI 1 *Staphylococcus aureus* (SA) for the indicated time. Cell death was measured by released LDH. Bars represent the mean ± SEM from at least three independent experiments. **c** Wild-type mice were injected i.p with DMSO or HG for 30 min and then infected with SA. CFU in lung and BAL were assayed 24 h after infection. **d** H&E histology of the representative lungs from mice treated with DMSO + PBS, HG + PBS, DMSO + SA, HG + SA for 24 h. Photomicrographs of histology are shown at ×100 magnification. **e–i** Relative mRNA levels of *Il-6*, *Tnf, Kc*, *Il-1β*, and *Cxcl2* from mice in (**b**) were assessed by RT-PCR. **j**, **k** Protein levels of TNF and IL-1β in serum from mice in (**b**). Results are representative of at least three independent experiments. Error bars represent SEM., Student’s *t* test, *p < 0.05.

Besides TNF-induced SIRS model, necroptosis also contribute to *Staphylococcus aureus*-mediated lung damage. It has been reported that *Staphylococcus aureus* can trigger necroptosis by injecting toxins [38]. Therefore, we sought to evaluate whether HG-9-91-01 decreases *Staphylococcus aureus*-mediated lung damage. Indeed, macrophages treated with HG-9-91-01 overcome cell death induced by *Staphylococcus aureus* and showed decreased RIPK3 auto-phosphorylation and MLKL phosphorylation (Fig. 8a, b). Mice treated with HG-9-91-01 presented lower lung damage and bacterial burden in their lung tissues and bronchoalveolar lavage fluid (BAL) compared to the control mice (Fig. 8c, d). Induction of pro-inflammatory cytokine and chemokine expression was also compromised in mice treated with HG-9-91-01 (Fig. 8e–k). Overall, our data suggest that HG-9-91-01 protects mice from *Staphylococcus aureus*-mediated lung damage.

## Discussion

HG-9-91-01 was used as an efficient inhibitor targeting SIKs (SIK1, SIK2 and SIK3), which has been employed in lots of works [32]. In previous studies, HG-9-91-01 was shown to directly target SIKs to promote downstream de-phosphorylation of transcriptional co-activators CRTC2/3, which results in enhanced relevant gene expression [39]. HG-9-91-01-interrupted SIKs-CRTC signaling pathway has been shown to participate in murine experimental colitis, melanin production, gluconeogenesis and β cell proliferation [32, 33, 39–43]. In our study, we discovered HG-9-91-01 as a RIPK3 kinase inhibitor in vitro and in vivo. Therefore, additional work needs to do that excludes the possibility that HG-9-91-01 may bypass SIKs to inhibit RIPK3 kinase activity to functions in murine experimental colitis, melanin production, gluconeogenesis andβcell proliferation.

Besides cellular protein brakes, several RIPK3 inhibitor including a well-characterized inhibitor GSK’872 were developed to interfere with necroptosis and necroptosis-related diseases. Our study demonstrated that HG-9-91-01 exhibited similar efficacy in inhibition of necroptosis compared with GSK’872. Besides inhibition of necroptosis, HG-9-91-01 and GSK’872 also induced RIPK3-dependent apoptosis in L929 cells at high concentrations. Knockdown of RIPK1 and caspase 8 exhibited resistance to HG-9-91-01-induced apoptosis. Unexpectedly, we also observed that depletion of caspase 1 decreased HG- or GSK’872-induced cell death and caspase 3 activation. Consistently, some previous works also reported the function of that caspase 1 to initiate apoptosis [44, 45]. Therefore, our data showed that caspase 1 participated in RIPK3 inhibitors-mediated apoptosis. Further work is needed to investigate how Caspase-1 participates in RIPK3-mediated apoptosis, and whether caspase-1 KO mice can rescue perinatal lethality of *Ripk3*^*D161N/D161N*^ mice. In addition, HG-9-91-01 could activate cleavage of GSDME and knockdown GSDME showed resistance to HG-9-91-01-induced cell death. Therefore, our work indicated that inhibition of RIPK3 mediated by HG-9-91-01 made cells sensitive to RIPK1-RIPK3-Caspase 1-Caspase 8-GSDME dependent pyroptosis. Similar results were obtained from mice carried RIPK3 kinase-dead D161N mutant, which drives Caspase 8 and RIPK1 dependent embryonic lethality [28]. However, mice expressing RIPK3 kinase inactive K51A mutant showed viable and fertile [27]. These data implied that kinase activity is not the determined step for RIPK3 dependent apoptosis. Kinase dead mutant (D161N) or high concentration kinase inhibitors show an impact on conformation that could unleash RHIM signaling and lead to caspase 8 dependent apoptosis. Although HG-9-91-01 and GSK’872 could trigger apoptosis in L929 cells, they could not induce significant cell death in HT29 and THP-1 human cells, which still inhibit human RIPK3 kinase activity and block necroptosis in HT29 cells. Therefore, further investigation needs to confirm and explain that why RIPK3 inhibitor do not trigger apoptosis in human cells.

The necrosome complex composed of RIPK1, RIPK3 and MLKL is a critical part for the regulation of necroptosis. Upon stimulation of necroptosis triggers, RIPK1 occurs as auto-phosphorylation and recruits RIPK3 through its RHIM domain, which leads to RIPK3 auto-phosphorylation and oligomerization and phosphorylation of downstream MLKL. In our study, we found that HG-9-91-01 diminished the association between RIPK3 and MLKL by directly targeting kinase activity. However, HG-9-91-01 enhanced the interaction of RIPK1 and RIPK3 and also indirectly promoted RIPK1 auto-phosphorylation. As RIPK1 inhibitor Nec-1 could not rescue the cell death induced by HG-9-91-01, increased auto-phosphorylation of RIPK1 did not contribute to HG-9-91-01-induced apoptosis. Although HG-9-91-01 could block the kinase activity of RIPK3, HG-9-91-01 also induced RIPK3 conformational changes and triggered RHIM-dependent oligomerization. Maybe the enhanced association of RIPK1 and RIPK3 is due to HG-9-91-01-induced RIPK3 conformational changes.

With the growing and deeper studies, RIPK3 are found to exhibit diverse functions in cell death and inflammation [25]. Our data demonstrated that HG-9-91-01 could induce RIPK1-RIPK3-Caspase 8-dependent pyroptosis by targeting RIPK3 kinase activity. Moreover, HG-9-91-01 also promoted TNF or TNF plus SM164 induced apoptosis in L929 cells or macrophages. Our results implied that HG-9-91-01 switched TNF-induced cell death from RIPK1-RIPK3-MLKL dependent necroptosis to RIPK3-MLKL-Caspase 1-Caspase 8 dependent apoptosis in L929 cells by blocking RIPK3 kinase activity and inducing RIPK3 proapoptotic conformation. Although HG-9-91-01 might promote cell apoptosis, HG-9-91-01 protected mice against TNF-induced SIRS and *Staphylococcus aureus*-mediated lung damage by inhibiting RIPK3-mediated necroptosis in vivo. Our data highlights that HG-9-91-01 can ben potential therapeutic target for treatment of necroptosis-mediated inflammatory injury based on inhibition of RIPK3 kinase activity. However, RIPK3 kinase inhibitor including HG-9-91-01 and GSK’872 triggered-apoptosis and -pyroptosis represents a challenge to the development of anti-inflammatory therapies targeting RIPK3. Considering this effect, combination of RIPK3 kinase inhibitor with apoptosis inhibitor may be better for treatment of necroptosis mediated inflammatory diseases. Moreover, further work may be needed to modify these chemicals, such as GSK’872 or HG-9-91-01, to avoid inducing apoptosis though RIPK3 conformation changes that unleashes RHIM-dependent oligomerization. On the other hand, aggressive induction of apoptosis or pyroptosis by RIPK3 inhibitors HG-9-91-01 we revealed here may have specific utility in cancer chemotherapy. Based on the multifaceted function of RIPK3 kinase inhibitors, further work need to be done, especially for functional verification in vivo.

## Supplementary information

Supplementary Figure and Table

uncropped western blots

Reproducibility checklist

## Data Availability

All data that support this study are available from the corresponding author on reasonable request.
