# Supplementary material for: Salt-inducible kinases inhibitor HG-9-91-01 targets RIPK3 kinase activity to alleviate necroptosis-mediated inflammatory injury
Source: Cell Death Dis. 2022 Feb 25;13(2):188. doi: 10.1038/s41419-022-04633-y (PMC8881470; doi:10.1038/s41419-022-04633-y)
Supplement: Supplementary file 1 — Supplementary Figure and Table [file 41419_2022_4633_MOESM1_ESM.doc]

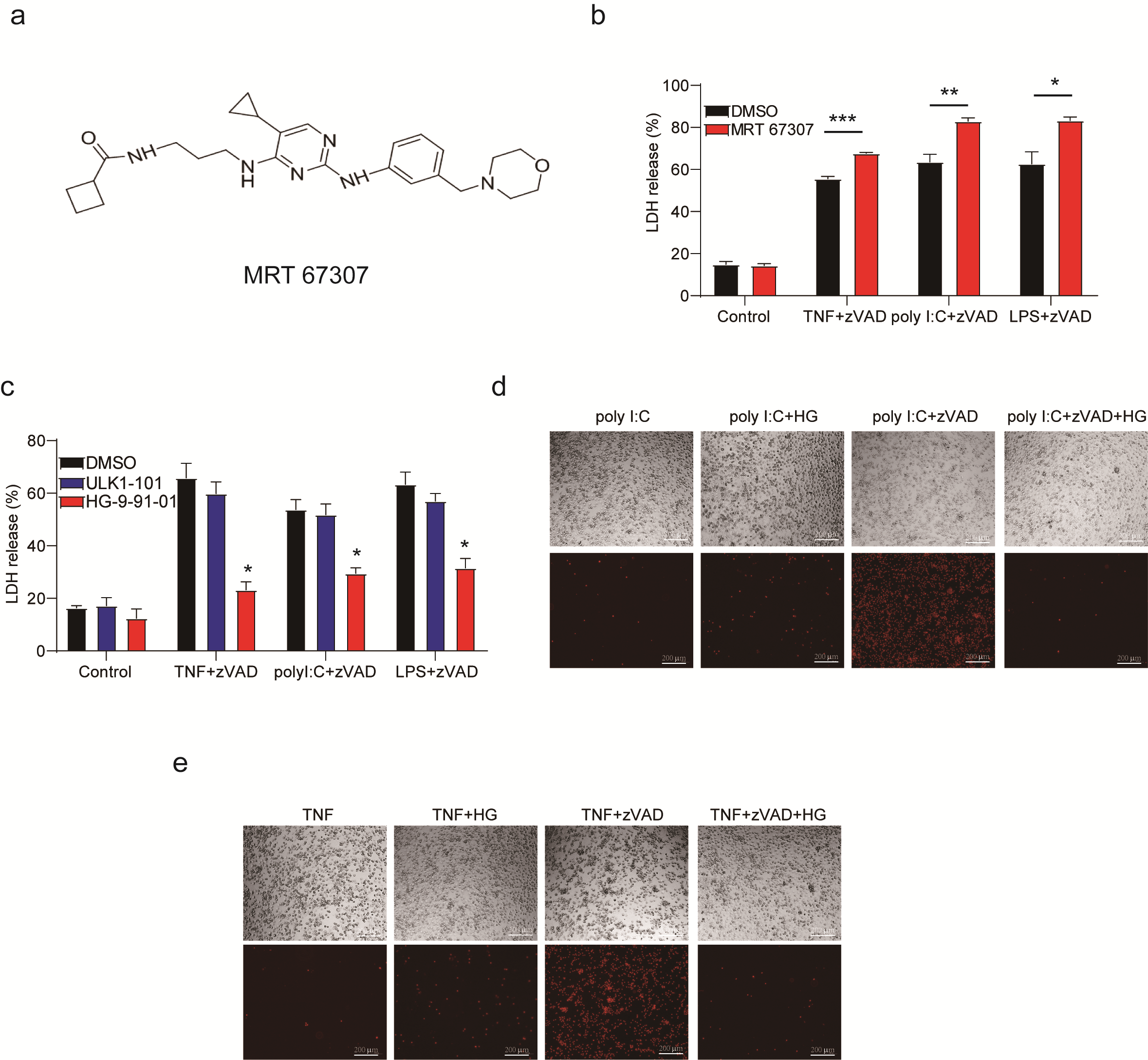


**Supplementary Figure 1. HG-9-9-01 inhibited TNF- and TLR-induced necroptosis in macrophages.**

**(a)** Chemical structure of MRT 67307. **(b)** Peritoneal macrophages were pretreated with MRT 67307 for 30 min and then treated with indicated ligands and compounds for 12 hours. Cell death was determined by released LDH.**(c)** Peritoneal macrophages were pretreated with 1 μM ULK or 1 μM HG for 30 min and then treated with indicated ligands and compounds for 12 hours. Cell death was determined by released LDH. **(d)** Peritoneal macrophages were pretreated with HG for 30 min and then treated with poly I:C or zVAD for 12 hours. Cells were stained with PI. Photomicrographs of histology were shown at 100× magnification. **(e)** Peritoneal macrophages were pretreated with HG for 30 min and then treated with TNF or zVAD for 12 hours. Cells were stained with PI. Photomicrographs of histology were shown at 100× magnification. Bars represent the mean ± SEM from at least three independent experiments. Student’s t-test *p < 0.05, **p < 0.01 and ***p< 0.001.

**
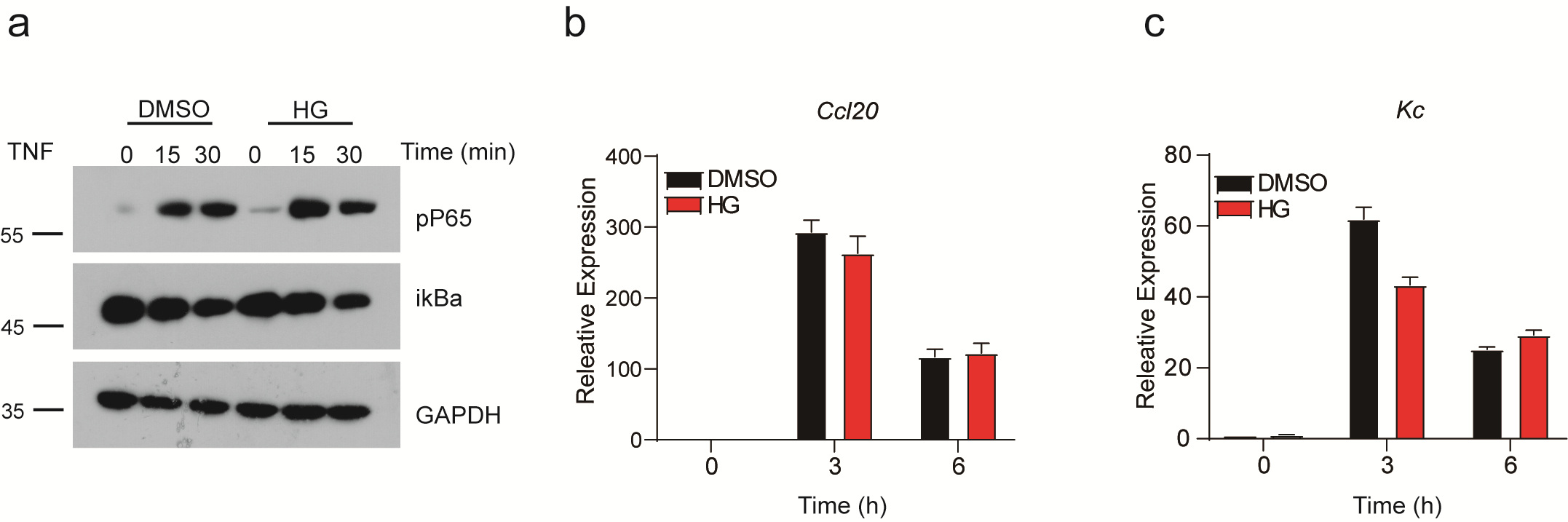
**

**Supplementary Figure 2. HG-9-9-01 showed no effect on TNF-induced downstream NF-κB activation.**

**(a)** HT29 cells were pretreated with HG-9-9-01 for 30 min and then stimulated with TNF for indicated time. Cell lysates were immunoblotted with indicated antibodies. **(b, c)** HT29 cells were pretreated with HG-9-9-01 for 30 min and then stimulated with TNF for indicated time. Quantitative mRNA level of *Ccl20* and *Kc* were analyzed by RT-PCR. Bars represent the mean ± SEM from at least three independent experiments.


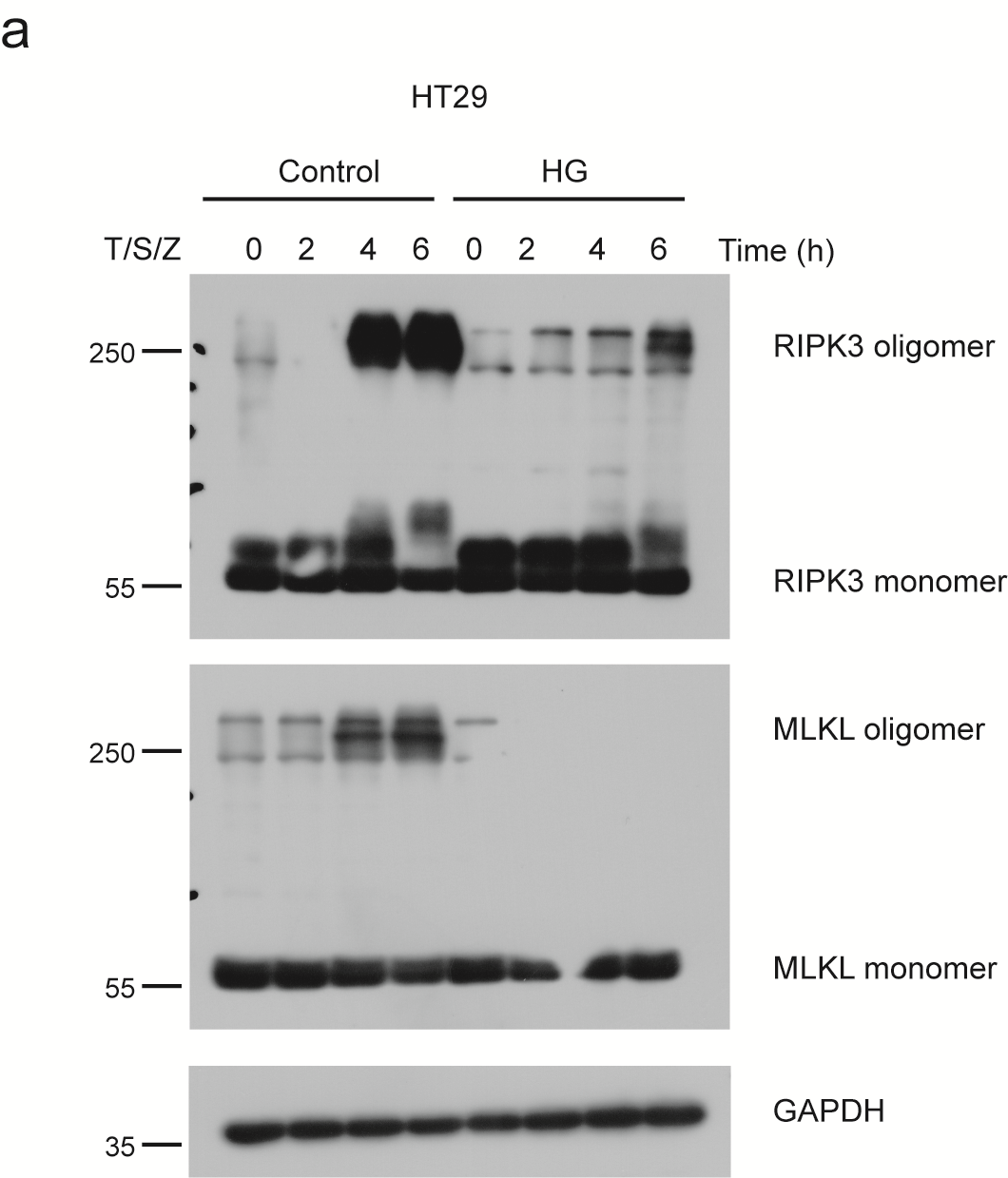


**Supplementary Figure 3. HG-9-9-01 blocked oligomerization of RIPK3 and MLKL in HT29 cells.**

**(a)** HT29 cells were pretreated with HG-9-9-01 for 30 min and then stimulated with TNF+SM164+zVAD for indicated time. Cell lysates were immunoblotted with indicated antibodies.


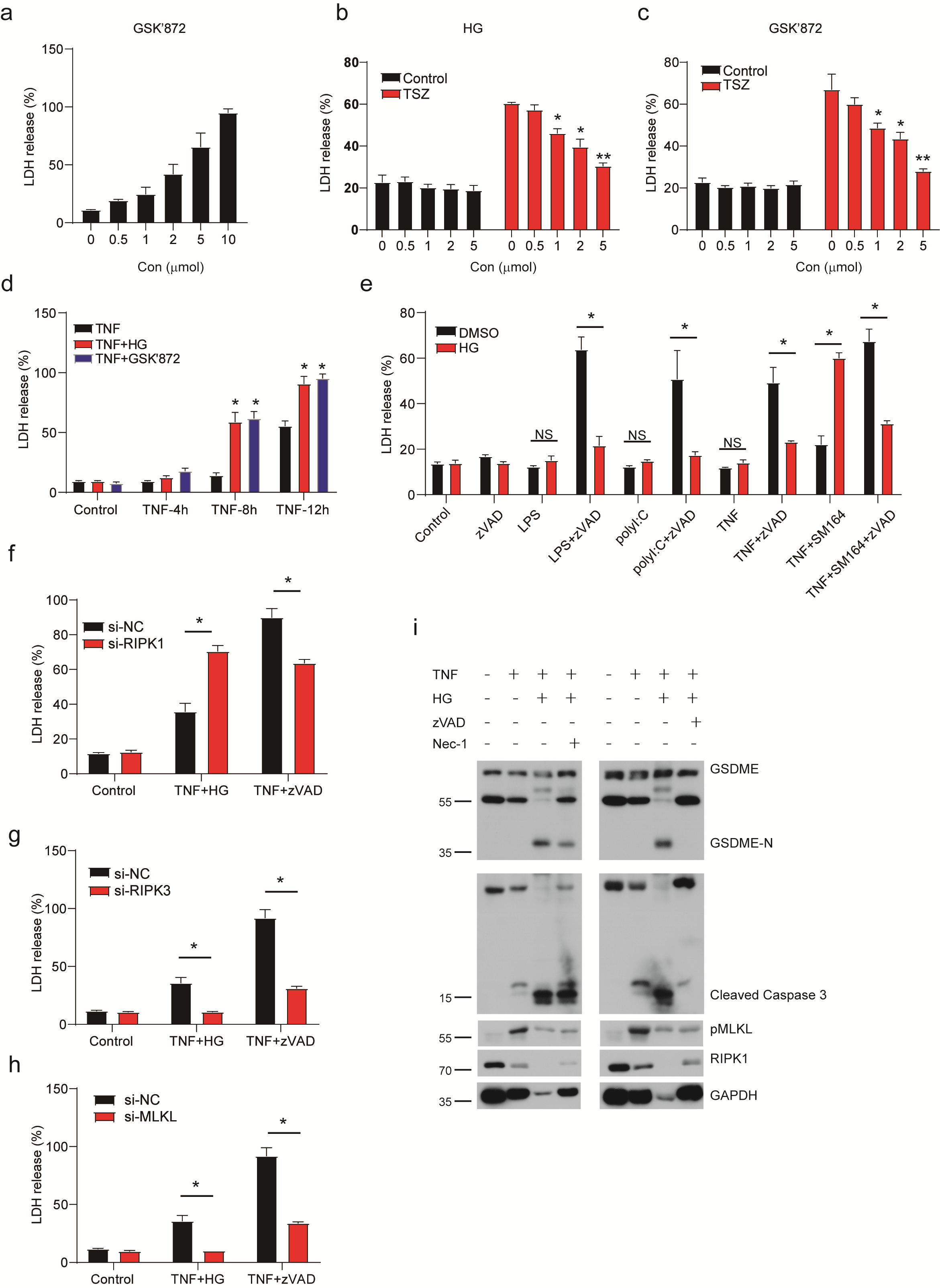


**Supplementary Figure 4. HG-9-9-01 promoted TNF-induced pyroptosis, which required RIPK3 and MLKL.**

**(a)** L929 cells were treated with GSK’872 at indicated concentration for 12 hours, cell death was determined by released LDH. **(b, c)** HT-29 cells were pretreated with HG-9-9-01 **(b)** or GSK’872 **(c)** for 30 min and then stimulated with TSZ for 12 hours. Cell death was measured by released LDH. **(d)** L929 cells were pretreated with HG-9-9-01 (0.25 μM) or GSK’872 (0.25 μM) for 30 min and then stimulated with TNF for indicated time. Cell death was measured by released LDH. **(e)** Peritoneal macrophages were pretreated with HG-9-9-01 (1 μM) for 30 min and then stimulated with indicated ligands or compounds for 12 hours. Cell death was measured by released LDH. (**f-h**) L929 cells were transfected with control siRNA, siRNA targeting RIPK1 **(f)**, siRNA targeting RIPK3 **(g)** or siRNA targeting MLKL **(h)** for 3 days. Then cells were treated with TNF plus HG or TNF plus zVAD for 12 hours. Cell death was determined by released LDH. **(i)** L929 cells were treated with indicated compounds and ligands. Cell lysates were immunoblotted with indicated with antibodies. Bars represent the mean ± SEM from at least three independent experiments. *p < 0.05, **p < 0.01 and ***p< 0.001.


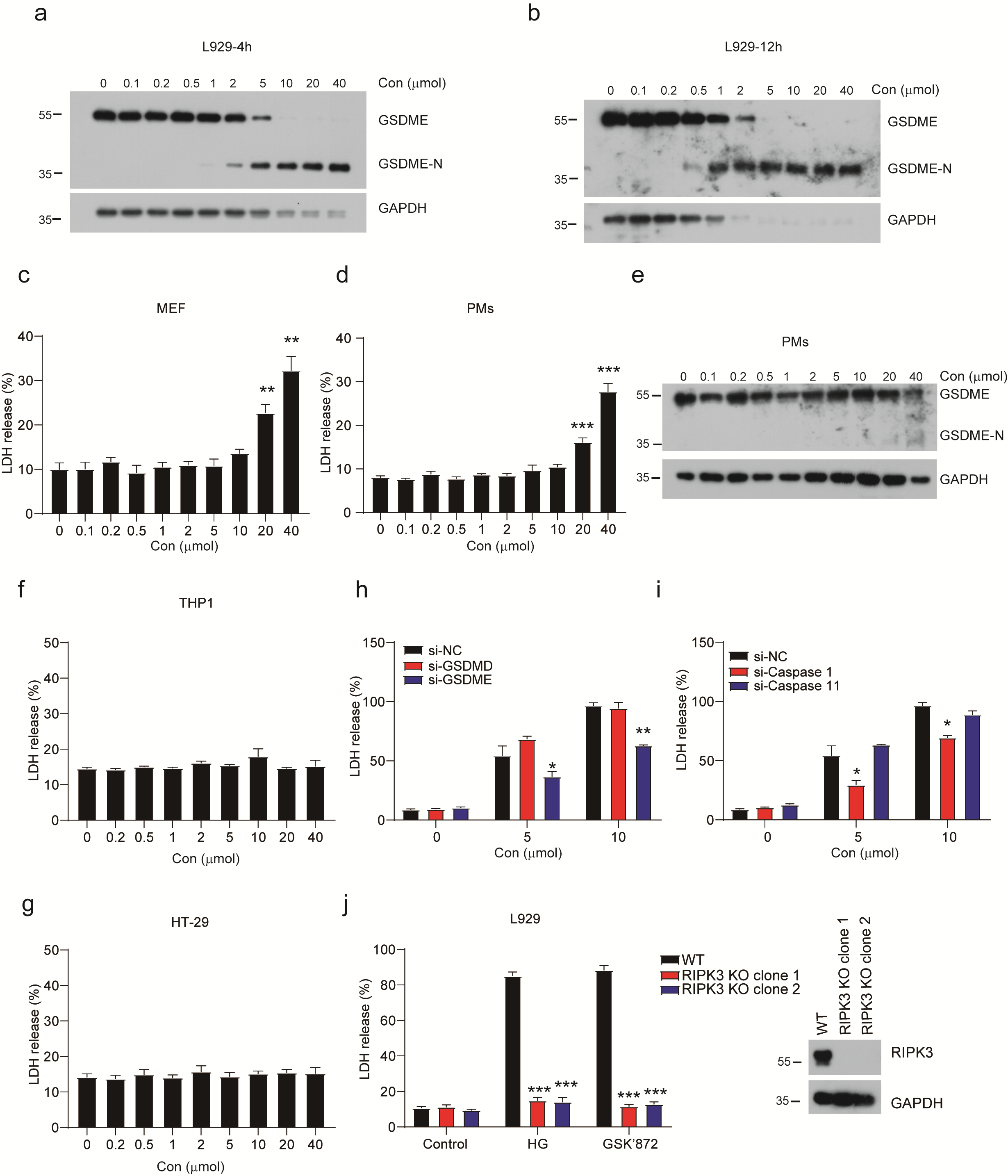


**Supplementary Figure 5. HG-9-9-01 and GSK’872 triggered RIPK3-RIPK1-Caspase 1-Caspase8-GSDME-dependent pyroptosis in L929 cells.**

**(a,b)** L929 cells were treated with different concentrations of HG-9-9-01 for 4h hours **(a)** and 12 hours **(b)**. Cell lysates were immunoblotted with indicated antibodies. **(c, d)** MEF cells **(c)**, and peritoneal macrophages **(d)** were stimulated with different concentrations of HG-9-9-01 for 12 hours. Cell death was determined with released LDH. **(e)** PMs were treated as in **(d),** cell lysates from PMs were immunoblotted with indicated antibodies. **(f, g)** THP1 and HT-29 cells were stimulated with different concentrations of HG-9-9-01 for 12 hours. Cell death was determined with released LDH. **(h, i)** L929 cells were treated with indicated siRNA oligo for 3 days and then treated with GSK’872 for 6 hours. Cell death was determined by released LDH.**(j)** The indicated L929 cells were challenged with 5 μM HG-9-9-01 and 10 μM GSK’872 for 12 hours. Cell death was measured by released LDH. RIPK3 ablation in RIPK3 knockout (KO) cells were confirmed by immunoblot.

**Supplementary Table 1**

| **Gene** | **Forward primer** | **Reserve primer** |
| --- | --- | --- |
| *Rpl13a* | GGGCAGGTTCTGGTATTGGAT | GGCTCGGAAATGGTAGGGG |
| *Sik1* | TGGACGTCTGGAGCCTCGGT | AGAGTGGGGTCGGCCTGCAT |
| *Sik2* | TGAGCAGGTTCTTCGACTGAT | AGATCGCATCAGTCTCACGTT |
| *Sik3* | TCCCCACTTGTCACCATGAC | GAGCGATGCTGGTCAGGTAC |
| *Crtc3* | TTGACCAACAGCCCATGA | GGTGGCTCTGCTGTACCAA |
| *Gsdme* | TGCAACTTCTAAGTCTGGTGACC | CTCCACAACCACTGGACTGAG |
| *Il-6* | GATGGATGCTACCAAACTGGAT | CCAGGTAGCTATGGTACTCCAGA |
| *Tnf* | TCTTCTCATTCCTGCTTGTGG | GGTCTGGGCCATAGAACTGA |
| *Kc* | AGACTCCAGCCACACTCCAA | TGACAGCGCAGCTCATTG |
| *Il-1β* | TGTAATGAAAGACGGCACACC | TCTTCTTTGGGTATTGCTTGG |
| *Cxcl2* | CCTGGTTCAGAAAATCATCCA | CTTCCGTTGAGGGACAGC |
| *Ccl20* | TGTACGAGAGGCAACAGTCG | TCTGCTCTTCCTTGCTTTGG |
