# Supplementary material for: Salt-inducible kinases inhibitor HG-9-91-01 targets RIPK3 kinase activity to alleviate necroptosis-mediated inflammatory injury
Source: Cell Death Dis. 2022 Feb 25;13(2):188. doi: 10.1038/s41419-022-04633-y (PMC8881470; doi:10.1038/s41419-022-04633-y)
Supplement: Supplementary file 2 — uncropped western blots [file 41419_2022_4633_MOESM2_ESM.doc]

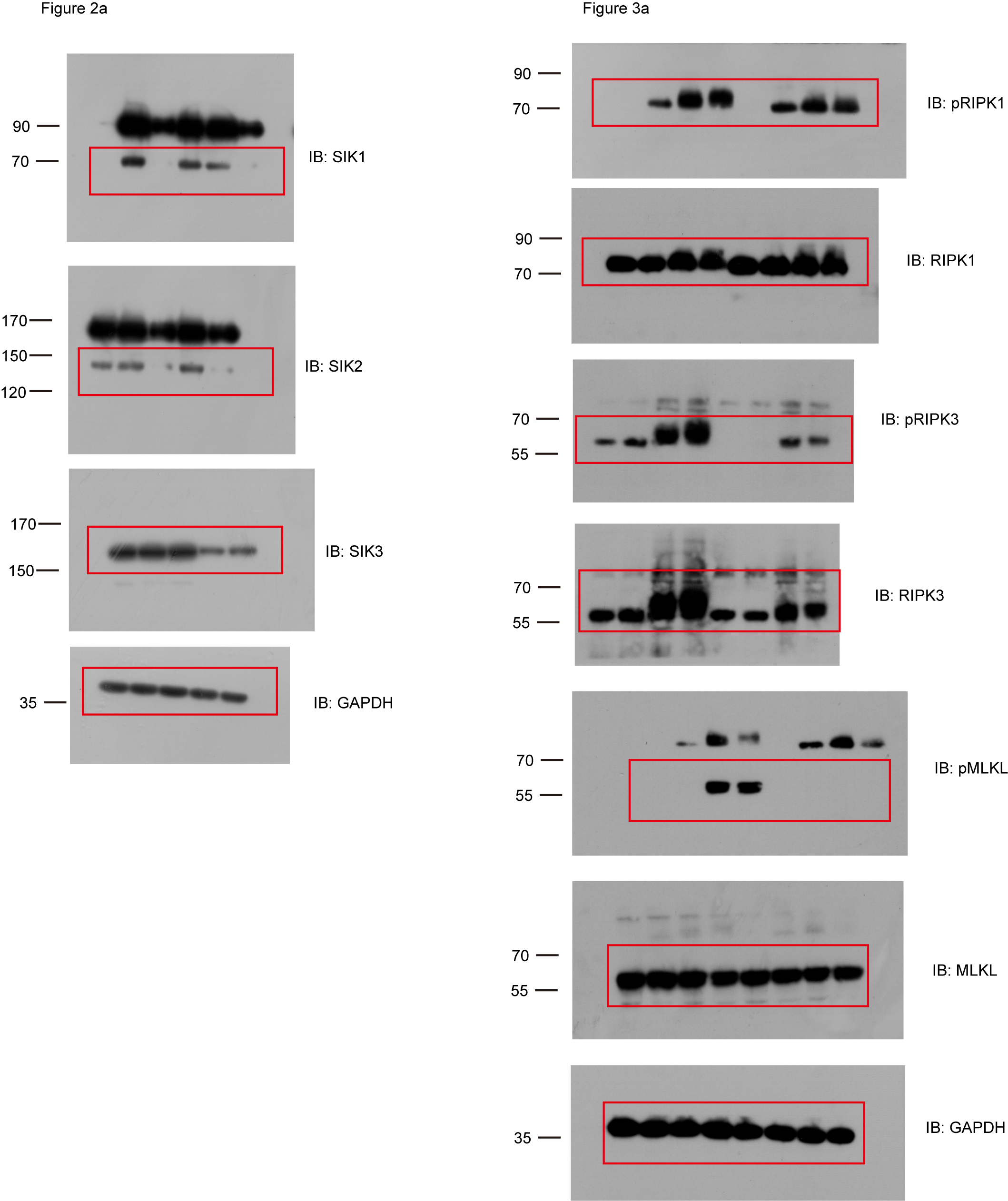


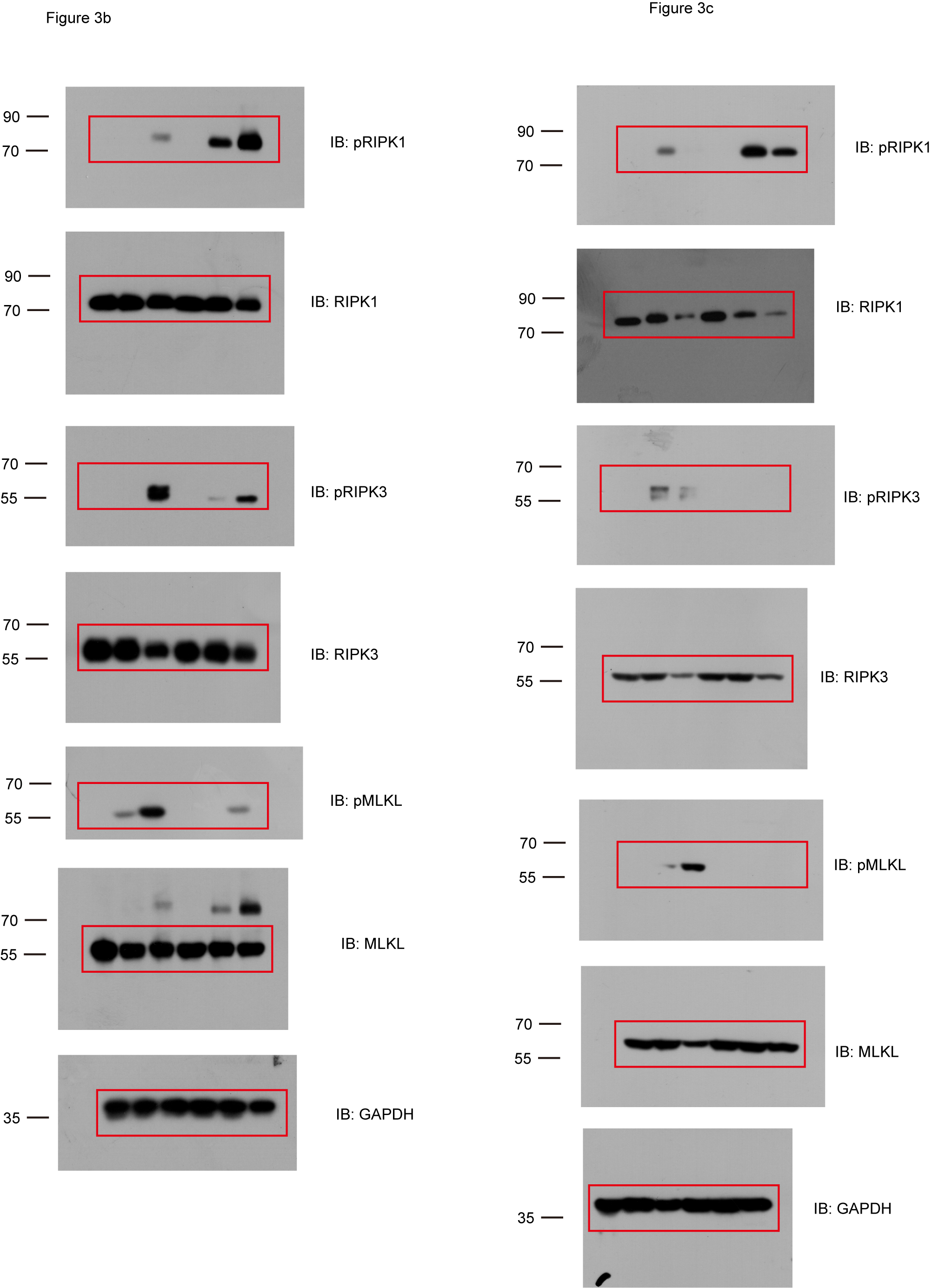

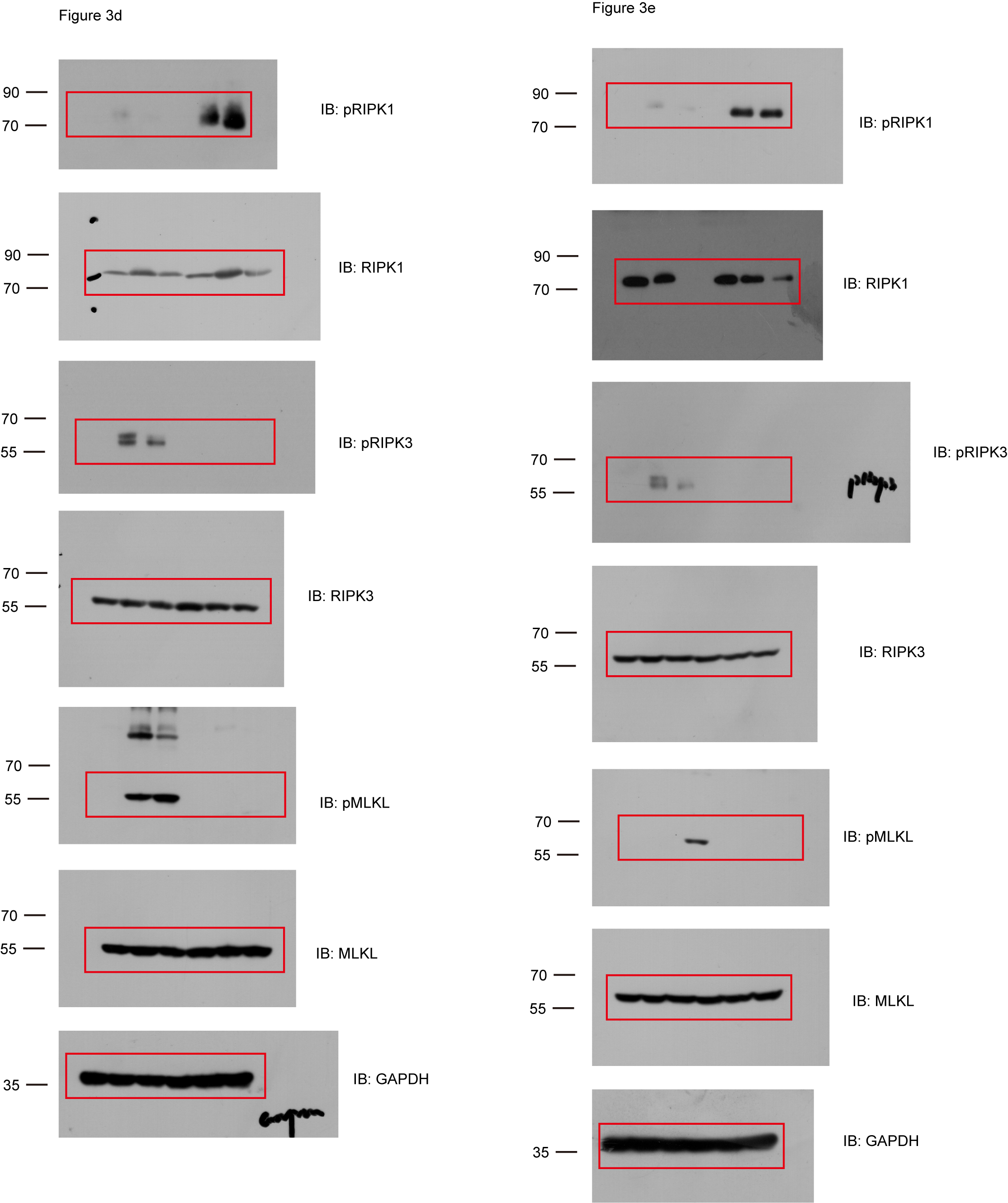

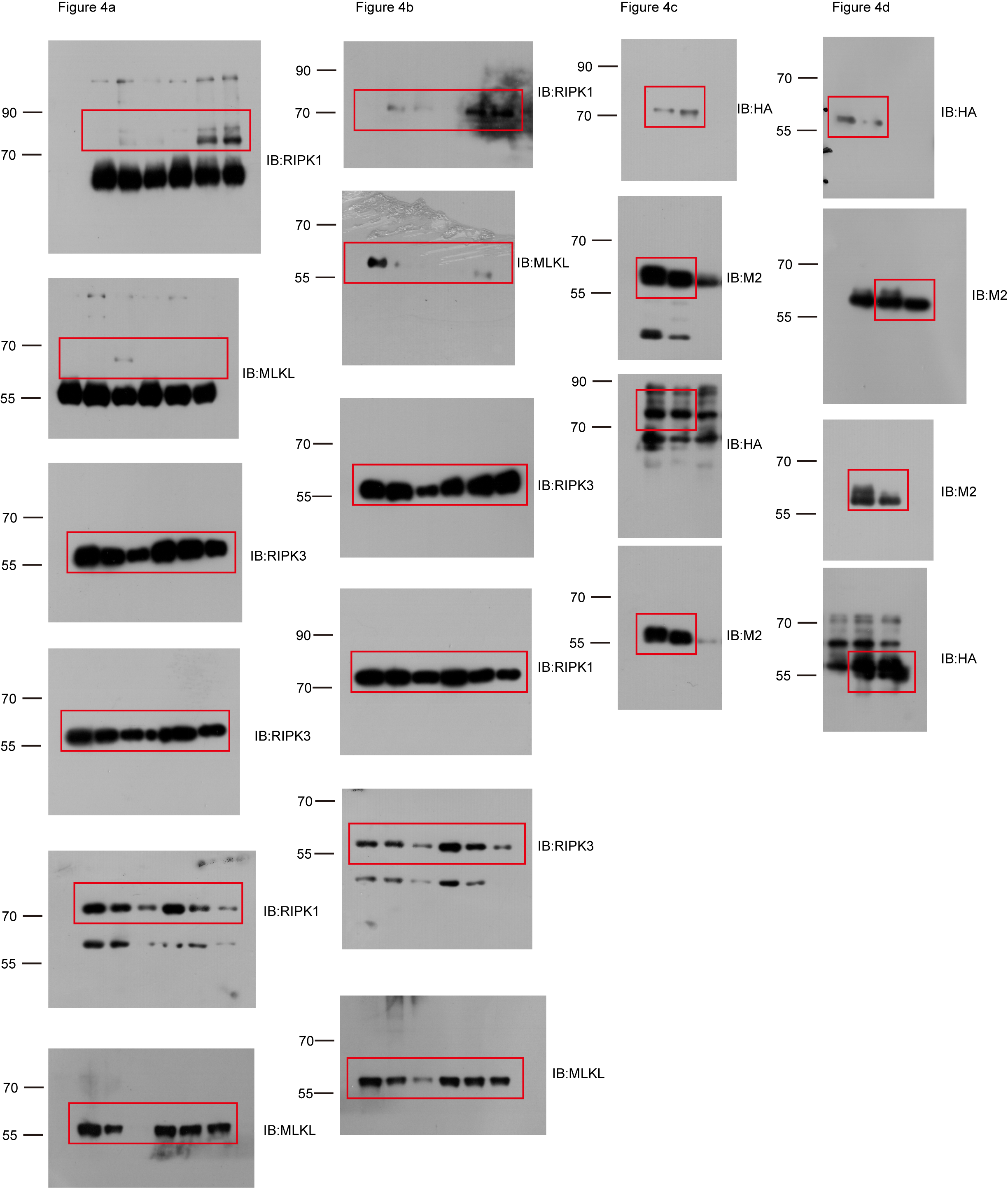

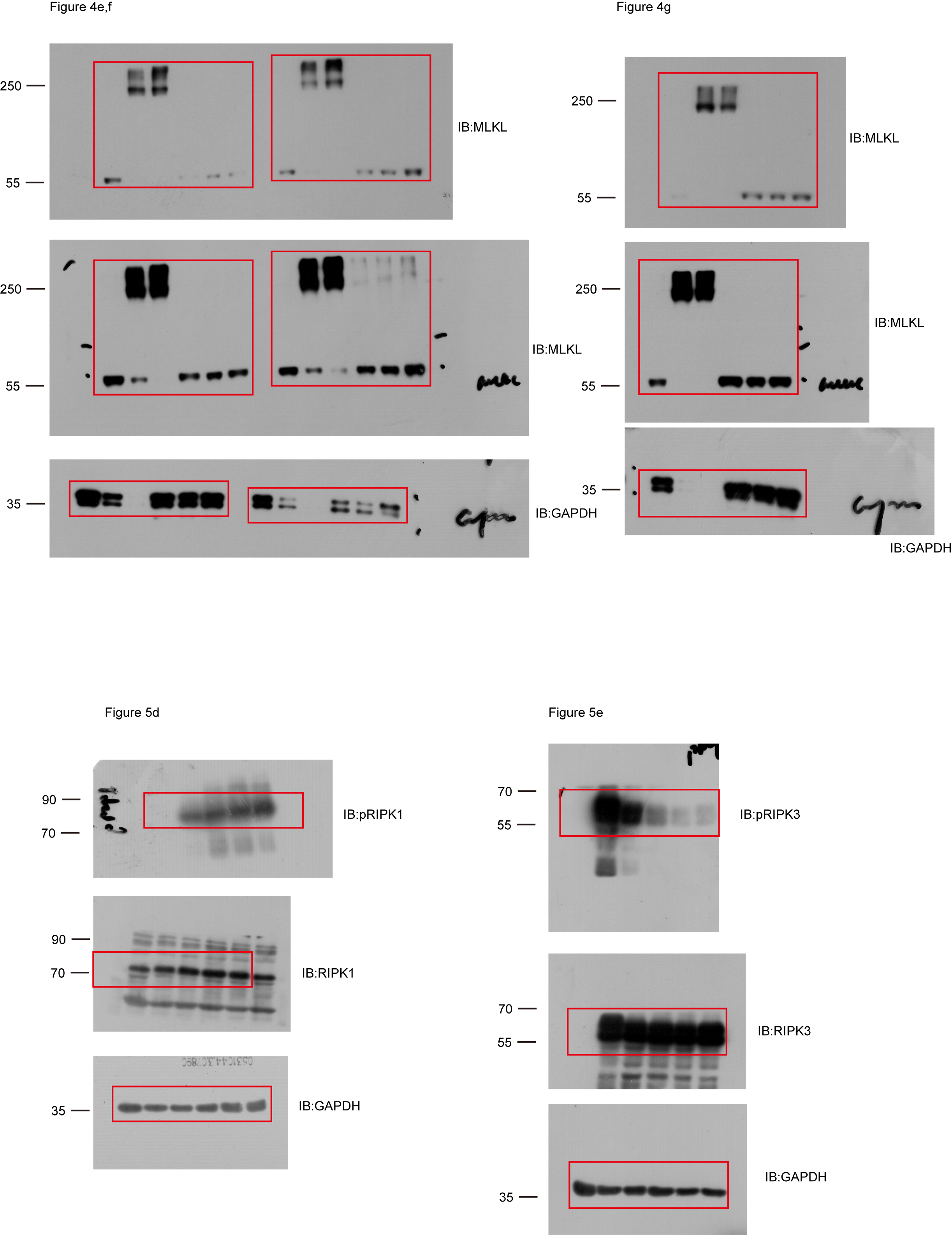

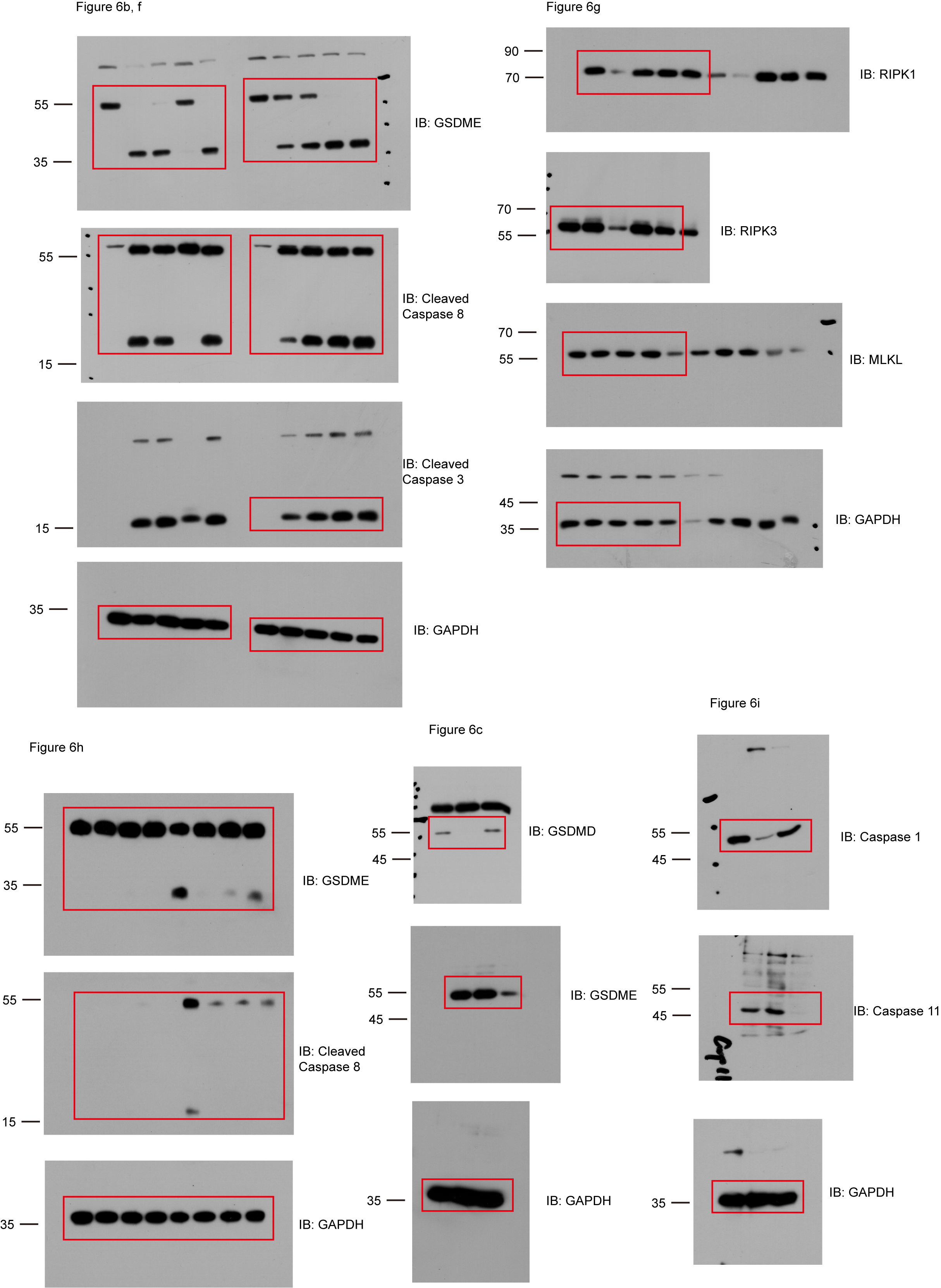

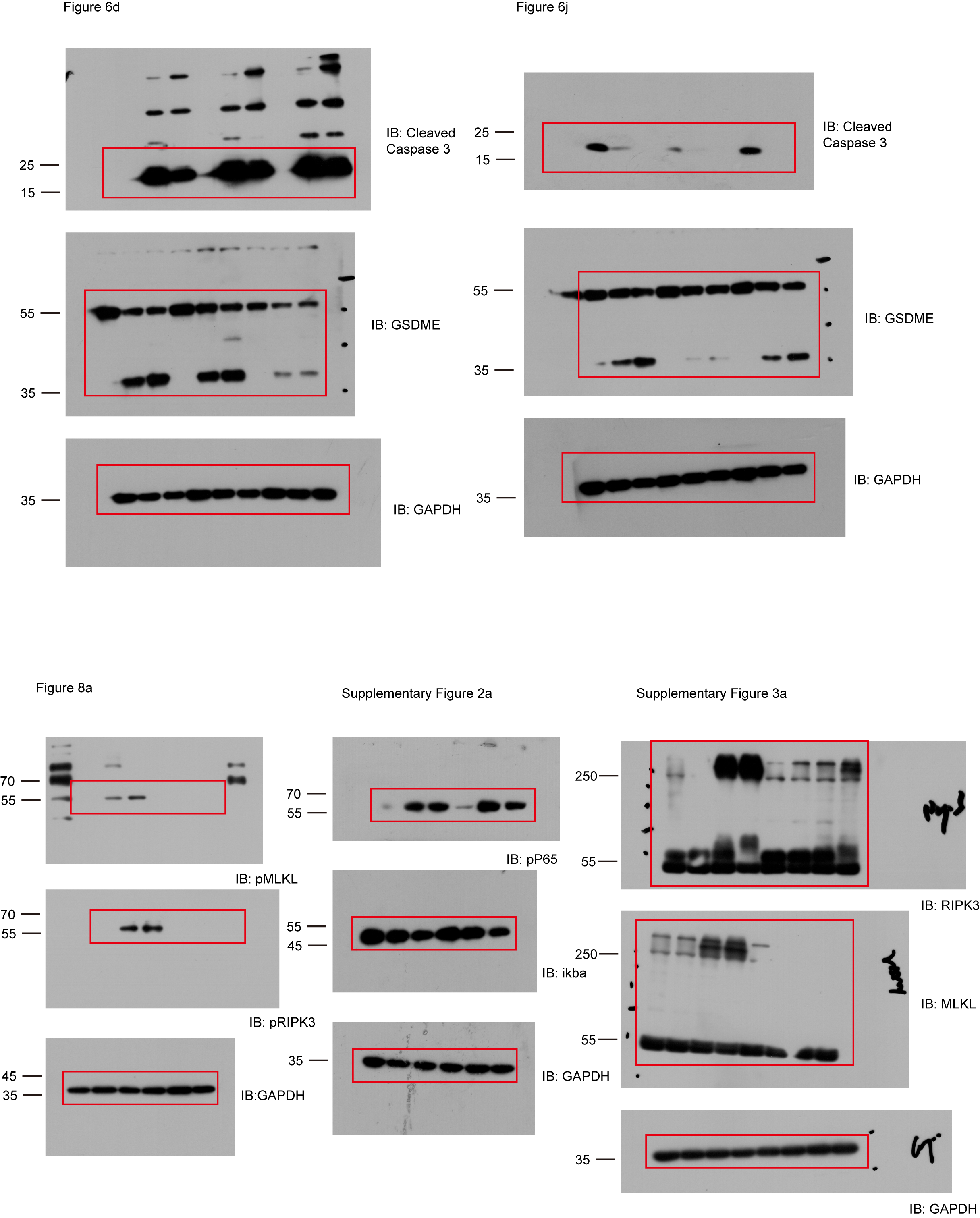

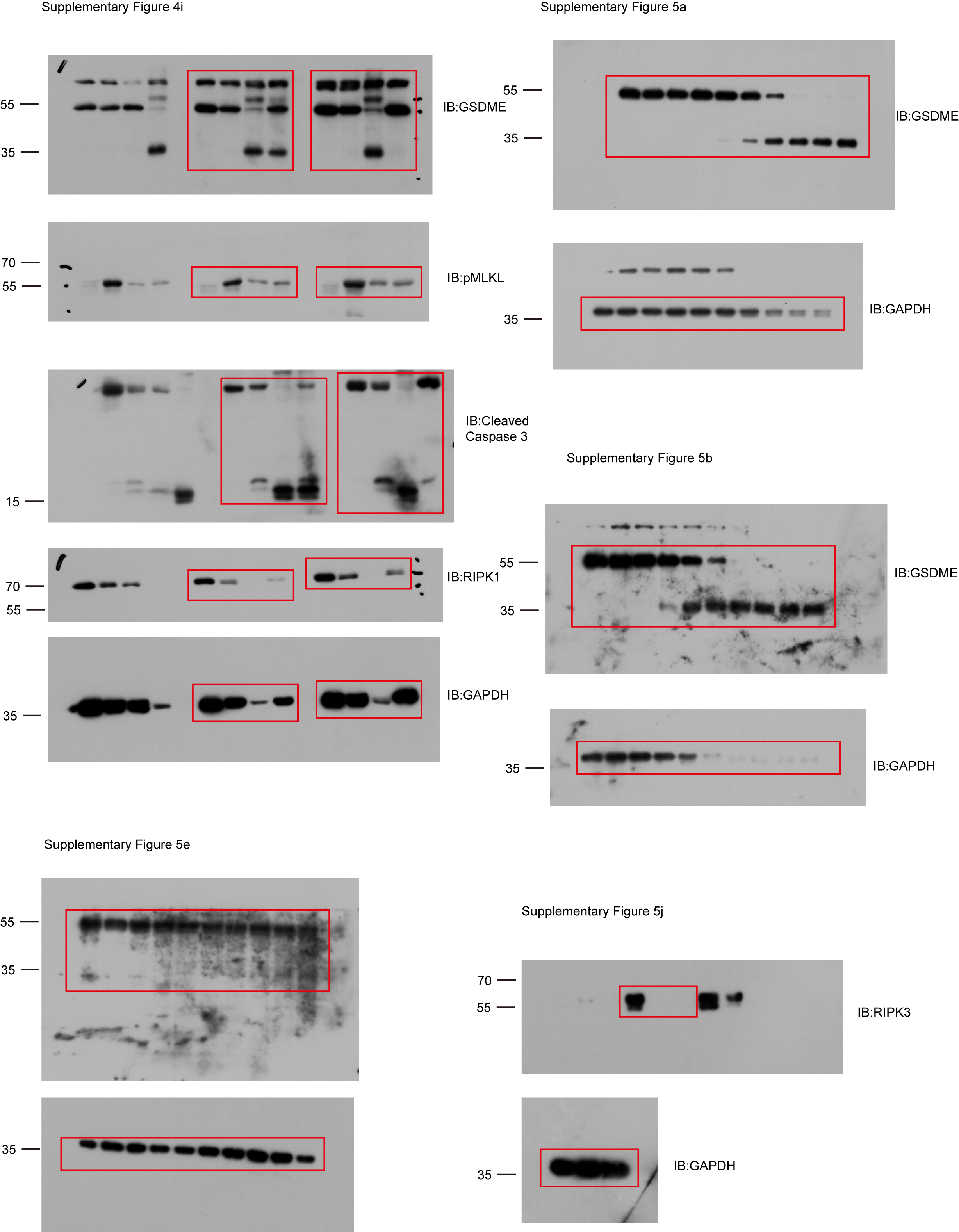


**Supplementary data**: Non-cropped versions of the western blots shown in Figure 2a, Figure 3a, 3b, 3c, 3d, 3e, Figure 4a-g, Figure 5d, 5e, Figure 6b, 6c, 6d, 6f, 6g, 6h, 6j, Figure 8a, Supplementary Figure 2a, Supplementary Figure 3, Supplementary Figure 4i and Supplementary Figure 5a, 5b, 5e, 5j.
